# A Chiral Virus‐Like Nanoplatform That Broadly Mediates Antibiotic Delivery to the Brain for Bacterial Meningitis Therapy

**DOI:** 10.1002/advs.78317

**Published:** 2026-10-18

**Authors:** Qianqian Xu, Xuting Zheng, Huinan Tong, Ying Liu, Xiaoxi Fan, Miao Xu, Ziyan Zhang, Li Zhang, Mingyan Liu, Ting Du, Heran Li, Guojun Liu

**Affiliations:** ^1^ School of Pharmacy China Medical University Shenyang China; ^2^ Department of Neurosurgery the Fourth Affiliated Hospital of China Medical University Shenyang China; ^3^ Department of Infectious Disease The First Hospital of China Medical University Shenyang China; ^4^ Department of Thoracic Surgery The First Hospital of China Medical University Shenyang China

**Keywords:** bacterial meningitis, blood‐brain barrier, cefoperazone, chiral mesoporous silica, imipenem, virus‐mimicking morphology

## Abstract

Blood‐brain barrier (BBB) preserves brain homeostasis yet restricts therapeutics; in bacterial meningitis, antibiotics’ limited penetration impairs delivery to the parenchyma and cerebrospinal fluid, constraining efficacy and motivating strategies to overcome the barrier. To address this, we developed a chiral virus‐mimicking mesoporous silica nanostructure (CVSN) functionalized with L‐alanine for antibiotic delivery. It offers several distinct advantages: (1) nanoscale dimensions facilitating BBB transport; (2) virus‐like morphology enhancing vascular accumulation and penetration, providing a foundation for passive targeting; (3) chiral surface modification conferring active recognition capacity for more efficient biological targeting; and (4) a mesoporous architecture that ensures high drug‐loading efficiency and controlled release of drugs. Experimentally, it achieves efficient BBB penetration into the brain parenchyma and shows targeted delivery into microglia and astrocytes. Simulating the loading of cargoes by viruses, it ultimately improves the bioavailability and brain parenchyma and cerebrospinal fluid contents of various antibiotics with distinct physicochemical properties, antibacterial spectrum, and BBB penetration capacity, including cefoperazone, norfloxacin, levofloxacin and imipenem. It helps to treat meningitis induced by both Gram‐negative bacteria (e.g., *Escherichia coli*) and Gram‐positive bacteria (e.g., *Staphylococcus aureus*) and provides a promising avenue to overcome the limitations imposed by the BBB for CNS infections antibiotic therapy in clinic.

## Graphical abstract

Chiral virus‐mimicking mesoporous silica nanostructures (CVSNs) efficiently transport diverse antibiotics across the blood–brain barrier (BBB) into brain parenchyma and cerebrospinal fluid. By enhancing delivery to microglia and astrocytes, CVSNs improve antibiotic bioavailability and therapeutic efficacy against both Gram‐negative and Gram‐positive bacterial meningitis, offering a promising strategy for CNS infection treatment.

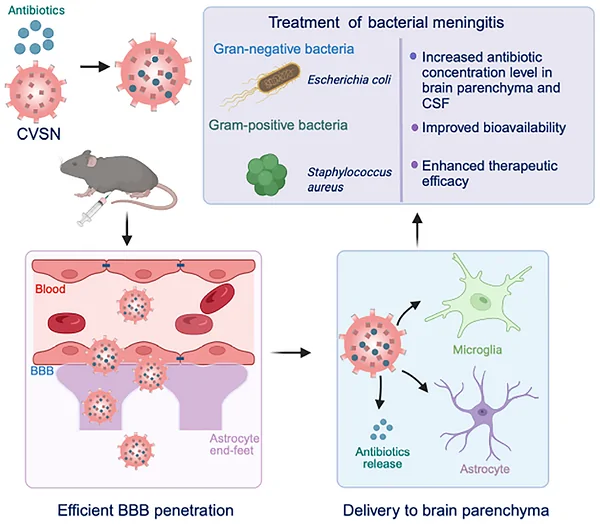

## 1 Introduction

Central nervous system (CNS) is the main regulatory and integrative site of the human body. Infectious disorders of the CNS frequently induce a cascade of intricate and severe pathological changes, significantly jeopardising human health and life safety [1]. Bacterial meningitis (BM) is one of the most common and fatal infections of the central nervous system (CNS) [2]. The worldwide number of new cases is close to 1.7 million annually with an estimated mortality rate ranging from 10% to 20% [3]. Presently, treatment of BM is dominated by antibiotics used on a conventional basis including third‐generation cephalosporins (ceftriaxone, cefoperazone) and vancomycin, which are antibiotics targeting common pathogens such as Streptococcus pneumoniae, Neisseria meningitidis, and Haemophilus influenzae [4]. Moreover, supportive management involves maintaining electrolyte balance, intracranial pressure control and the prevention or the management of shock [5]. Glucocorticoids (e.g., dexamethasone) are occasionally administered to attenuate inflammatory responses and lessen the likelihood of neurological sequelae [6]. Contemporary therapeutic approaches, however, do not improve patient outcomes, and the growing problem of antibiotic resistance makes conventional antibiotics less effective, presenting considerable challenges in controlling the disease [7].

The pathogenesis of bacterial meningitis (BM) is initiated when pathogen‐associated molecular patterns (PAMPs) cross the blood–brain barrier (BBB) and activate innate immune responses [8]. Gram‐negative bacteria release lipopolysaccharides (LPS/LOS), primarily triggering TLR4‐mediated signaling [9]. while Gram‐positive bacteria activate TLR2 pathways through peptidoglycan and lipoteichoic acids [10]. These processes converge on NF‐κB–dependent inflammatory cascades, leading to the release of pro‐inflammatory cytokines such as TNF‐α, IL‐1β, and IL‐6 [11]. In addition, bacterial toxins and DNA further amplify inflammation and promote inflammasome activation. The resulting immune response is characterized by extensive neutrophil infiltration and activation of microglia and macrophages, accompanied by the production of proteases, reactive oxygen species, and inflammatory mediators [12]. Together with factors such as MMP‐9 and VEGF, these events disrupt tight junctions, impair BBB integrity, and contribute to cerebral edema [13]. Although antibiotics remain the primary treatment for BM, their therapeutic efficacy is limited by the BBB [14]. Due to its tight junctions and selective transport systems, more than 98% of small‐molecule drugs and nearly all macromolecules cannot effectively penetrate into the brain [15]. Consequently, antibiotic concentrations in cerebrospinal fluid (CSF) are often insufficient, leading to suboptimal outcomes and increased risk of resistance [16]. Therefore, developing strategies to enhance targeted antibiotic delivery across the BBB is of critical importance in BM treatment.

The application of nanomaterials in biomedicine has expanded rapidly in recent years, owing to their high surface‐to‐volume ratio, tunable pore size, excellent biocompatibility, and facile functionalization [17]. In particular, nanomaterial‐based strategies have emerged as promising alternatives against bacterial infections; for instance, Wang et al. developed oxygenated carbon nanotubes with enhanced peroxidase‐mimicking activity that effectively eliminated bacterial pathogens both in vitro and in vivo, demonstrating the feasibility of nanozymes as robust antibacterial platforms [18]. Notably, nanomaterial‐based strategies have emerged as promising non‐antibiotic alternatives against bacterial infections; for example, Bao, Hu, Liu et al. reported highly crystalline copper aluminum‐layered double hydroxides (CuAl‐LDHs) with intrinsic Fenton‐like catalytic activity, which efficiently generated ·OH radicals in the presence of H_2_O_2_ and achieved broad‐spectrum catalytic sterilization against both Gram‐positive and Gram‐negative pathogens [19]. Considerable progress has been made in the development of novel nanomedicines for the treatment of bacterial meningitis [20]. For instance, Shi et al. engineered a fully biomimetic magnetic outer membrane vesicle (OMV) system carrying ceftriaxone and the sonosensitizer tetraphenylporphyrin, which could be magnetically guided across the BBB to facilitate sonodynamic therapy of bacterial meningitis [21]. Chen et al. [22] exploited the BBB‐invasive capacity of *Escherichia coli* K1 outer membranes to achieve brain‐targeted delivery. They constructed biomimetic self‐assembled nanoparticles decorated with *Escherichia coli* K1 outer membrane lipooligosaccharides, enabling BBB penetration through a transcytotic vesicular transport pathway. Hong et al. designed a mixed micelle system comprising polyethylene glycolylated bacteriocin A nanoparticles, a brain‐targeting peptide (rabies virus glycopeptide 29, RVG29), and a P‐glycoprotein inhibitor (Pluronic P85 monomer). This system demonstrated potent antibacterial activity against 13 clinically isolated Streptococcus pneumoniae strains, with enhanced uptake by brain capillary endothelial cells (BCECs) and superior BBB penetration [23]. In addition, Wenping Zhu et al. developed artificial bacteriophage‐mimicking nanostructures that enabled localized bacterial capture and enhanced catalytic sterilization, providing a new paradigm for biomimetic antibacterial nanoplatforms against infectious diseases [24]. Liu et al. reported virus‐like copper silicate hollow spheres as artificial bacteriophages, in which the spiky surface topography shortened the distance between nanoparticles and pathogens to enable localized bacterial capture and Fenton‐like catalytic sterilization for treating oral infectious diseases, demonstrating the promise of virus‐mimicking nanostructures in antibacterial applications [25]. Despite these advances, the translational development of biomimetic nanoplatforms requires a balance among structural sophistication, fabrication reproducibility, physicochemical stability, manufacturability, and therapeutic performance. In the present study, we therefore focus on the functional integration of virus‐mimicking topology, chiral surface modification, and mesoporous drug loading, together with experimental evaluation of the physicochemical properties and biological performance of the resulting platform.

In recent years, chiral nanoplatforms have emerged as promising delivery systems in biomedicine, particularly for diseases requiring precise biological targeting [26]. Because living systems are inherently chiral, the introduction of chirality into nanomaterials can significantly affect their interactions with biomolecules, cell membranes, and receptors. Compared with conventional achiral carriers, chiral nanoplatforms offer several notable advantages: they can improve stereoselective recognition and cellular internalization, enhance specificity toward target cells, modulate in vivo biodistribution and retention, and potentially facilitate transport across complex biological barriers such as the blood‐brain barrier [27]. In addition, when integrated with porous nanostructures, chiral modification can be combined with high drug‐loading capacity and controlled release behavior, thereby improving therapeutic bioavailability at the disease site [28]. These advantages suggest that chiral nanocarriers may provide a valuable strategy for enhancing antibiotic delivery to the central nervous system and overcoming the limitations of conventional treatments for bacterial meningitis.

L‐alanine was selected as the surface chiral ligand based on both biomimetic and physicochemical considerations [29]. First, as a naturally occurring endogenous amino acid, L‐alanine has favorable biocompatibility and low structural complexity, which makes it suitable for surface engineering without introducing excessive steric hindrance or compromising the virus‐like morphology and mesoporous architecture of the nanocarrier. Second, its small molecular size and accessible amino/carboxyl functionalities facilitate stable covalent conjugation while preserving the hydrophilic interfacial properties required for vascular transport. Third, because biological interfaces are inherently chiral, the L‐enantiomer was expected to provide more physiologically relevant stereochemical interactions with membrane‐associated proteins and cellular components than non‐chiral or unnatural surface ligands, thereby promoting nano‐bio recognition and uptake [29, 30]. In addition, L‐alanine was used as a rational benchmark chiral ligand to distinguish the contribution of surface chirality from that of particle shape alone, which was further supported by comparison with D‐alanine‐modified contrsols [31]. On the basis of these considerations, L‐alanine was ultimately chosen as a simple, biocompatible, and biologically relevant chiral moiety to endow the virus‐like nanoplatform with enhanced BBB translocation and cellular internalization capacity.

Building on this design rationale, we developed a chiral virus‐mimicking mesoporous silica nanoparticle delivery system to overcome BBB limitations and improve the treatment of BM. It employs a multi‐contact viral spike‐like structure functionalized with biomimetic L‐alanine ligands to enhance BBB penetration, enabling specific distribution in the brain parenchyma, ventricles, and meninges. Once inside the CNS, the nanocarrier achieves controlled release of antibiotics, including cefoperazone (CPZ), norfloxacin (NOR), levofloxacin (LFX), and imipenem (IPM), thereby effectively eliminating pathogens and inhibiting bacterial proliferation. Moreover, by releasing antibiotics targeting the choroid plexus epithelium, the nanocarrier alleviates inflammatory injury and preserves barrier function, further suppressing pro‐inflammatory cytokine release and leukocyte infiltration, ultimately mitigating meningeal inflammation and cerebral edema. Furthermore, its prolonged circulation profile maintains therapeutic drug concentrations in the CSF and brain tissue, reducing dosing frequency and systemic toxicity, thereby improving both patient compliance and safety.

## 2 Results

### 2.1 Synthesis and Characterization of CVSN

We employed a biphasic reaction system consisting of cetyltrimethylammonium bromide (CTAB) and tetraethyl orthosilicate (TEOS). By exploiting the unique properties of the oil‐water interface and the templating effect of single micelles, nanoparticles were directed to self‐assemble along specific orientations into virus‐shaped nanoparticles (VSN). Subsequently, VSN were subjected to amination, followed by acylation of their surface amino groups with Boc‐L‐alanine, enabling the grafting of amino acids onto the particle surface. This process ultimately yielded chiral modified virus‐shaped nanoparticles (CVSN) (Figure 1A). Transmission electron microscopy (TEM) and scanning electron microscopy (SEM) analyses revealed no significant differences in morphology or size between VSN and CVSN, which both exhibited a typical virus‐like architecture with uniformly distributed nanoscale spikes (Figure 1B). The particle size distributions of VSN and CVSN were highly uniform, with average diameters maintained within the range of 40–50 nm (Figure 1C,E), contributing to consistent performance. Fourier‐transform infrared (FTIR) spectroscopy confirmed the internal siloxane framework of the nanoparticles, as evidenced by Si‐O‐Si stretching and bending vibration peaks at 3300 and 470 cm^−1^ respectively. After amination, VSN‐NH_2_ and CVSN exhibited an N‐H stretching vibration peak at 1450 cm^−1^, confirming the successful introduction of amino groups onto the particle surfaces. Upon L‐alanine grafting, CVSN showed a characteristic amide bond peak at 1650 cm^−1^, further validating the occurrence of the grafting reaction (Figure 1D). To further evaluate the chemical stability of CVSN, FTIR spectra were collected for freshly synthesized CVSN, CVSN after 5 months of storage, and CVSN recovered after 7 days of serum incubation. The major characteristic absorption bands associated with the siloxane framework and alanine‐derived surface functionalities were retained in all three samples, without obvious loss of the principal spectral features (Figure S1). These results support preservation of the main chemical structure of CVSN during long‐term storage and after prolonged exposure to a protein‐rich biological environment. X‐ray photoelectron spectroscopy (XPS) was further employed to investigate the surface elemental composition and chemical modification of the nanoparticles (Figure 1F). The survey spectra of both VSN and CVSN exhibited characteristic Si 2p, C 1s, and O 1s signals at approximately 103, 284.8, and 532–533 eV, respectively, consistent with the silica‐based framework and surface carbon‐containing species. Notably, CVSN exhibited an additional N 1s signal at approximately 399–400 eV, which was absent in VSN. The emergence of this nitrogen signal after surface functionalization indicates the introduction of nitrogen‐containing organic groups associated with the L‐alanine‐modified surface. Together with the characteristic amide‐related band observed by FTIR and the increased organic weight loss detected by TGA, the XPS results provide complementary evidence for the successful surface functionalization of VSN with L‐alanine. Furthermore, TEM mapping of CVSN revealed that Si constituted the highest proportion (76.38%), followed by C and O, while N accounted for 1.3%. The nitrogen content primarily originated from the amide bonds and the amino group of alanine itself (Figure 1G,M).

**FIGURE 1:**
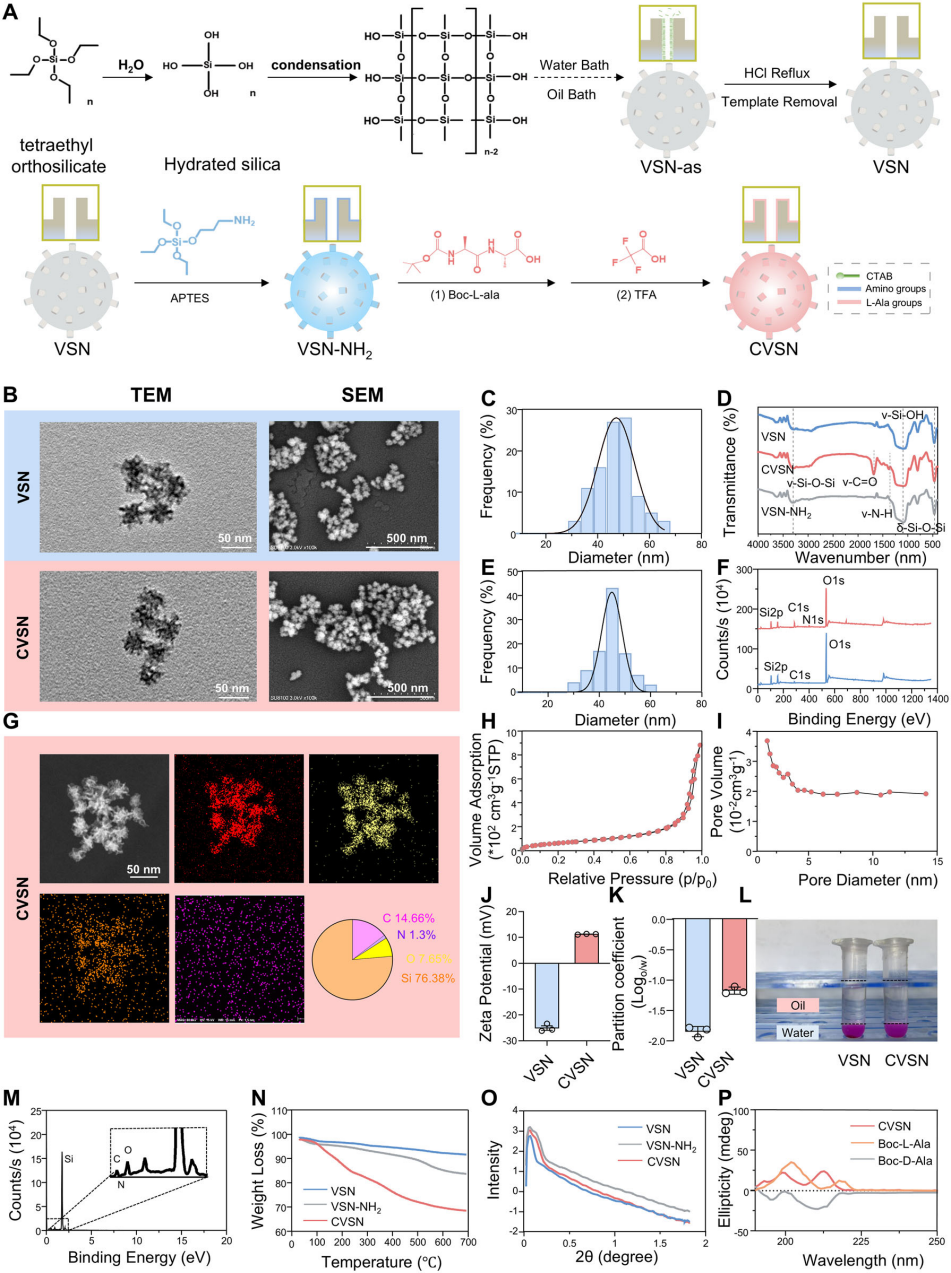
Biomimetic synthesis and characterization of CVSN. (A) Schematic illustration of the synthetic procedure of CVSN. (B) Transmission electron microscopy (TEM) and scanning electron microscopy (SEM) images of VSN and CVSN. (C) Particle size distribution of VSN (calculated from TEM images). (D) Fourier‐transform infrared (FTIR) spectra. (E) Particle size distribution of CVSN (calculated from TEM images). (F) X‐ray photoelectron spectroscopy (XPS) spectra. (G) TEM mapping images of CVSN. (H) Nitrogen adsorption‐desorption isotherms. (I) Pore size distribution curve of CVSN. (J) Zeta potential measurements. Data are presented as mean ± SEM, *n* = 3, n refers to the number of biological replicates. (K) Oil‐water partition coefficients. Data are presented as mean ± SEM, *n* = 3, n refers to the number of biological replicates. (L) Oil‐water partition images. (M) composition profile derived from TEM mapping. (N) TGA curves. (O) Small‐angle X‐ray scattering (SAXS) patterns. (P) Circular dichroism (CD) spectra.

Nitrogen adsorption‐desorption isotherms revealed a typical H1‐type hysteresis loop, characteristic of a type IV isotherm (Figure 1H). This result indicates that CVSN possess well‐defined mesoporous structures with uniform pore size distribution and ordered channels. Barrett‐Joyner‐Halenda (BJH) analysis further confirmed an average pore diameter of 3.37 nm (Figure 1I). Zeta potential measurements demonstrated that the surface potentials of VSN and CVSN were −28.23 ± 2.74 mV and +11.21 ± 0.13 mV, respectively (Figure 1J). Partition coefficient (log P) measurements yielded consistently negative values (Figure 1K), indicating that the nanoparticles are intrinsically hydrophilic, consistent with the results of the oil‐water distribution experiment (Figure 1L). This hydrophilic character is favorable for maintaining nanoparticle dispersibility and colloidal stability in aqueous physiological environments, thereby reducing aggregation during systemic circulation. It may also minimize nonspecific hydrophobic interactions with biological components, providing a favorable basis for intravenous administration and vascular transport. After grafting with chiral L‐alanine, the surface potential significantly increased, shifting from negative to positive values, indicating that the modification markedly altered the surface charge characteristics of the nanoparticles. Upon heating to 700°C, thermogravimetric analysis (TGA) revealed weight losses of 8.34%, 16.37%, and 31.55% for VSN, VSN‐NH_2_, and CVSN, respectively (Figure 1N). VSN exhibited the lowest weight loss due to the stability of its siloxane framework. In contrast, VSN‐NH_2_ displayed greater weight loss as a result of the decomposition of nitrogen‐containing organic groups introduced during amination. The most significant mass loss was observed in CVSN, attributable to the thermal decomposition of grafted L‐alanine. Based on these results, the grafting density of L‐alanine was estimated to be approximately 15.18%. Small‐angle X‐ray scattering (SAXS) analysis showed distinct diffraction peaks within the 0.1°–5° (2θ) range (Figure 1O), suggesting the presence of periodic internal structures. Both CVSN and their synthetic intermediates displayed ordered pore size distributions and stable framework structures. Circular dichroism (CD) spectroscopy revealed a positive Cotton effect within the 180–250 nm wavelength range for both CVSN and L‐alanine, indicating preferential absorption of left‐handed circularly polarized light (Figure 1P). In contrast, D‐alanine exhibited an opposite signal. This confirms that L‐alanine was successfully grafted onto the CVSN surface, conferring stable and consistent chiral characteristics that are advantageous for virus‐mimetic functionality.

To further highlight the advantages of L‐alanine‐modified virus‐shaped nanocarriers in subsequent experiments, conventional spherical mesoporous silica nanoparticles (MSN) and D‐alanine‐modified virus‐shaped nanoparticles (DVSN) were successfully synthesized as control groups. Their successful preparation was confirmed through a series of characterization analyses. TEM and SEM images (Figure S2) showed that MSN exhibited a typical spherical morphology with an average particle size of approximately 90 nm, whereas DVSN displayed a virus‐like architecture with a smaller average diameter of around 40 nm. These results indicate that virus‐shaped nanoparticles possess a distinct size advantage over traditional spherical MSN, while modification with either L‐ or D‐alanine does not significantly alter the particle size. TGA revealed that DVSN exhibited a weight loss of up to 36%, which was markedly higher than that of MSN and VSN‐NH_2_, indicating the successful grafting of D‐alanine onto the virus‐shaped nanoparticles (Figure S3A). FTIR spectroscopy showed a characteristic amide bond peak at 1650 cm^−^^1^, further confirming the successful conjugation of D‐alanine (Figure S3B). XPS analysis demonstrated the presence of Si, O, and C elements in both VSN and DVSN, while chlorine was detected exclusively in DVSN (Figure S3C), providing additional evidence for successful surface modification. CD spectroscopy revealed that both DVSN and D‐alanine exhibited a positive Cotton effect in the 180–250 nm wavelength range, indicating preferential absorption of right‐handed circularly polarized light (Figure S3D). These findings collectively confirm the successful synthesis and chiral functionalization of DVSN, supporting their use as an appropriate control for evaluating the unique properties of L‐alanine‐modified CVSN.

Furthermore, the stability and degradation behaviors of CVSN were assessed by incubation in phosphate‐buffered saline (PBS, pH = 7.4) and artificial cerebrospinal fluid (aCSF). In both media, CVSN exhibited similar and relatively slow degradation kinetics. Within the first 2 h, ∼30% degradation was observed in both PBS and aCSF. Over time, the degradation rate gradually slowed, reaching 78% in PBS and 69% in aCSF after two weeks (Figure S4). These results indicate that CVSN undergo progressive degradation under physiological conditions, thereby minimizing the risk of long‐term accumulation and reducing potential toxicity in vivo.

### 2.2 Brain Targeting and Biodistribution of CVSN

To elucidate the spatiotemporal distribution of CVSN in mice, we intravenously injected RITC‐CVSN via the femoral vein, followed by tissue collection at predetermined time points. Fluorescence distribution was examined using an in vivo imaging system and stereomicroscopy (Figure 2A). At 1 h post‐injection, detectable fluorescence signals were observed in all major organs (Figure 2B), indicating that RITC‐CVSN rapidly disseminated through the systemic circulation. A portion of nanoparticles accumulated in the lungs, likely due to passive targeting effects, while the strongest signal was detected in the liver, consistent with its role as the primary clearance organ. Notably, pronounced fluorescence was also detected in the brain (Figure 2C). The brain signals peaked at 2 h and then gradually declined, suggesting progressive clearance of CVSN. Although the signal further decreased at 12 h, residual fluorescence remained detectable. This prolonged cerebral retention may be attributable to the spiky nanotube surface of CVSN, which increases vascular endothelial contact area and adhesive interactions, thereby facilitating therapeutic action. In addition, small‐animal imaging experiments of DVSN and VSN were performed using the same method (Figure S5). The results showed that the nanoparticles were predominantly accumulated in the liver and lungs, while their content in the brain reached the highest level at 2 h post‐administration. These biodistribution characteristics were generally consistent with those observed for DVSN. Further comparison revealed that the nanoparticle accumulation in the brain followed the order of CVSN > DVSN > VSN, indicating that chirality could enhance the brain‐targeting capability of the nanocarriers, with CVSN exhibiting superior brain‐targeting efficiency compared with DVSN.

**FIGURE 2:**
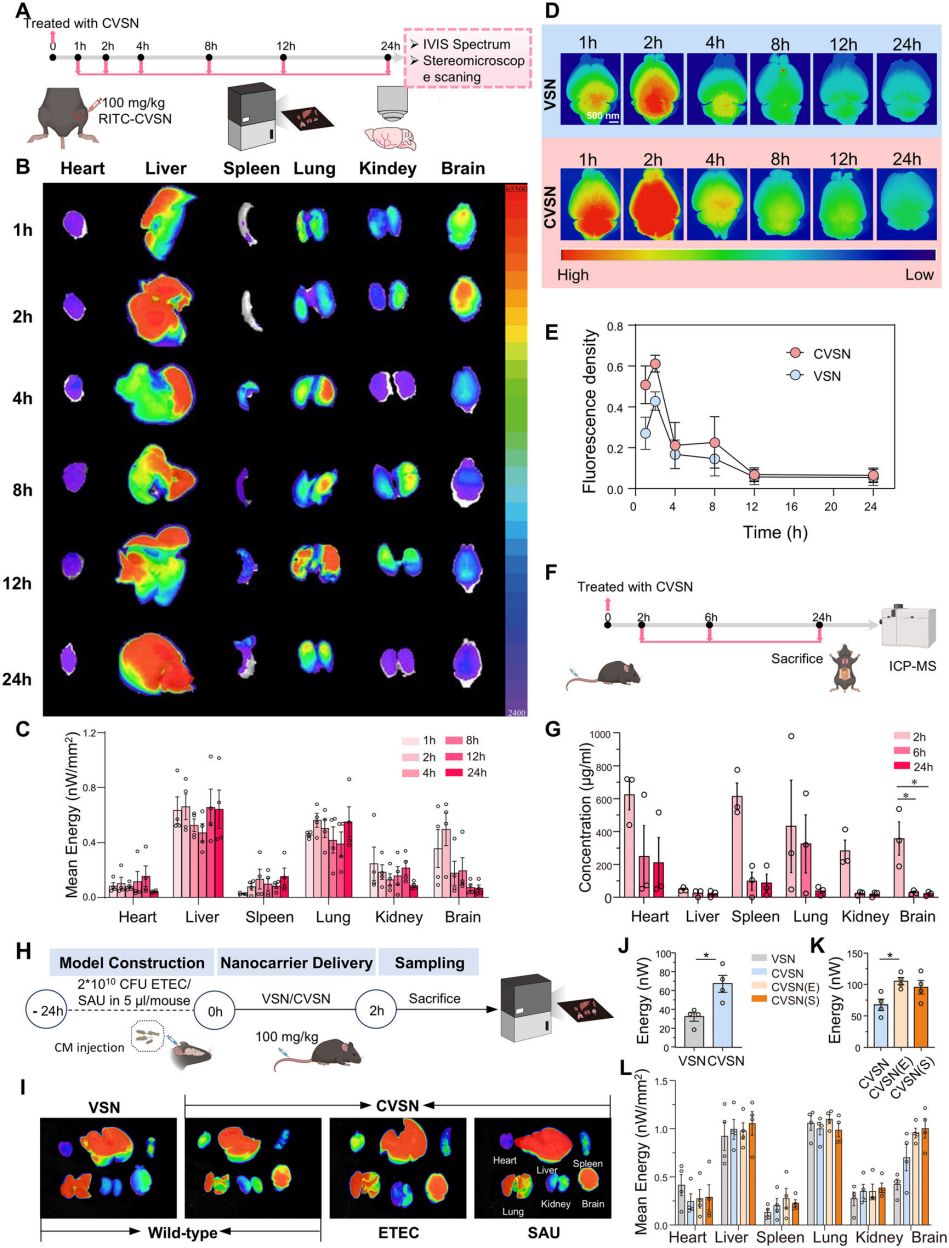
Tissue distribution of CVSN. (A) Schematic illustration of the experimental workflow for in vivo biodistribution of CVSN. (B) Representative whole‐body fluorescence imaging and (C) quantification of fluorescence intensity in major organs at different time points following intravenous administration of RITC‐CVSN in mice. Data are presented as mean ± SEM, *n* = 4, n refers to the number of biological replicates. (D) Stereomicroscopic images of brain tissue and (E) corresponding fluorescence intensity at different time points after intravenous injection of RITC‐CVSN and RITC‐VSN. Data are presented as mean ± SEM, *n* = 3, n refers to the number of biological replicates. (F) Schematic workflow of inductively coupled plasma mass spectrometry (ICP‐MS) analysis for silicon content in tissues and (G) quantitative Si levels at different time points post‐injection. Data are presented as mean ± SEM, *n =* 3, n refers to the number of biological replicates. (H) Schematic of the experimental procedure for evaluating brain‐targeted distribution in meningitis mouse models. (I) Fluorescence imaging of organs and tissues in vivo at 2 h after intravenous injection of RITC‐VSN/RITC‐CVSN in wild‐type and meningitis mice. (J) Fluorescence intensity in the brains of wild‐type mice following RITC‐VSN/RITC‐CVSN injection. (K) Brain fluorescence intensity in meningitis mice following RITC‐CVSN injection. (L) Fluorescence intensity per unit area in major organs. Data are presented as mean ± SEM, *n* = 3, n refers to the number of biological replicates. *p < 0.05. One‐way analysis of variance (ANOVA) post hoc Tukey's test was performed (G, K). Two‐sided unpaired t‐test was performed (J).

To further determine whether brain accumulation was dependent on the extent of L‐alanine surface functionalization, we prepared CVSN formulations using 0.5‐fold and 2‐fold L‐alanine feed levels relative to the standard CVSN formulation and evaluated their time‐dependent biodistribution in vivo (Figure S6). Both CVSN(0.5 × L‐Ala) and CVSN(2 × L‐Ala) exhibited systemic distribution after intravenous administration, with prominent fluorescence signals in the liver and lung and detectable accumulation in the brain. However, altering the L‐alanine functionalization level away from the standard formulation did not result in a sustained enhancement of brain accumulation. In particular, the lower‐functionalization formulation exhibited reduced brain fluorescence, whereas increasing the L‐alanine feed to 2‐fold did not provide a further improvement over the standard CVSN formulation. Together with the biodistribution profile of CVSN(1 × L‐Ala), these findings indicate a non‐linear relationship between L‐alanine surface functionalization and brain accumulation, suggesting that an intermediate level of ligand presentation is more favorable for brain targeting.

To further characterize the dynamic changes in the brain, stereomicroscopic imaging of brain tissues was performed, yielding results consistent with the observations described above. In addition, the intracerebral distribution of VSN at different time points was systematically investigated. The results showed that the fluorescence intensity of CVSN was consistently higher than that of VSN at all time points, directly demonstrating that chiral modification enhances accumulation and retention in the target organ. In other words, chiral modification significantly increases the brain accumulation of VSN (Figure 2D). Quantitative analysis further revealed that the fluorescence intensity reached its peak at 2 h and decreased to below 0.1 at 12 h. The peak fluorescence intensity was 0.61 for CVSN and 0.42 for VSN (Figure 2E). To exclude potential interference from fluorescence imaging, we quantified silicon (Si) content in tissues and organs using inductively coupled plasma mass spectrometry (ICP‐MS) (Figure 2F). At 2 h, most tissues exhibited elevated Si levels, which markedly decreased over time, reaching trace levels at 24 h. This trend was particularly evident in the brain, where Si content at 2 h was significantly higher than at 6 and 24 h (Figure 2G).

Infectious diseases of the brain are often accompanied by disruption of the BBB, with BM causing particularly pronounced damage [14, 32]. To preliminarily assess the brain‐targeting capacity of CVSN in a meningitis model, we first evaluated its distribution in brain tissue using in vivo fluorescence imaging in mice (Figure 2H). The results showed that both groups primarily accumulated in the liver and lungs, with brain signals ranking immediately after these organs (Figure 2I,L). In healthy mice, the fluorescence intensities of VSN and CVSN in the brain were 32.49 and 67.59 eV, respectively (Figure 2J). The significantly higher signal of CVSN compared to VSN was attributed to the chiral recognition ability conferred by L‐Ala grafting, which enhanced its targeting efficiency. In mice with *Escherichia coli* and *Staphylococcus aureus* meningitis, the brain fluorescence intensities of CVSN were 105.23 and 95.40 eV, respectively (Figure 2K), markedly higher than in healthy controls. This finding suggests that, under pathological conditions, the increased permeability of the BBB promotes greater penetration of CVSN into the brain, thereby facilitating the delivery of the loaded antibiotics to the lesion site and enhancing its antibacterial therapeutic efficacy.

Finally, to evaluate the fluorescence stability of RITC‐CVSN, we incubated the nanoparticles in two representative media—PBS (pH = 7.4) and aCSF—to mimic physiological microenvironments. RITC‐CVSN maintained stable fluorescence intensity over 24 h in both media (Figure S7), demonstrating excellent optical stability in vivo and ensuring reliable imaging and quantitative analysis.

### 2.3 Time‐Dependent BBB Penetration of CVSN

Based on the peak signal observed in in vivo imaging, brain tissues were collected at 2 h post‐injection. Mice receiving intravenous injections of RITC‐CVSN and the vascular marker lectin were perfused, and their brains were extracted and sectioned. Confocal laser scanning microscopy (CLSM) and whole‐slide imaging were then employed for analysis (Figure 3A). Representative brain sections from anterior, middle, and posterior regions were subjected to full‐field scanning across three fluorescence channels (Figure 3B). The mean fluorescence intensity of RITC‐CVSN was quantified in the dorsal cortex (D‐CTX), lateral cortex (L‐CTX), ventral cortex (V‐CTX), hippocampus (HC), thalamus (T), and hypothalamus (HT) in the middle sections. The results showed no significant regional differences, indicating a uniform distribution of RITC‐CVSN within the brain without preferential accumulation in specific areas (Figure 3C,D). Comparison of overall fluorescence intensity across sections revealed that the anterior (including striatum) and middle (including hippocampus) levels exhibited similar signals, both slightly lower than those in the posterior sections (which encompassed a broader hippocampal region) (Figure 3E,F). The whole‐slide scanning results of MSN and DVSN brain sections (Figure S8) further demonstrated a homogeneous distribution across different brain regions, with no significant regional differences observed. This distribution pattern is consistent with the intrinsic characteristics of VSN. Notably, the fluorescence intensity of DVSN was significantly higher than that of MSN, suggesting that the virus‐mimicking nanostructure confers a distinct advantage in traversing the blood–brain barrier.

**FIGURE 3:**
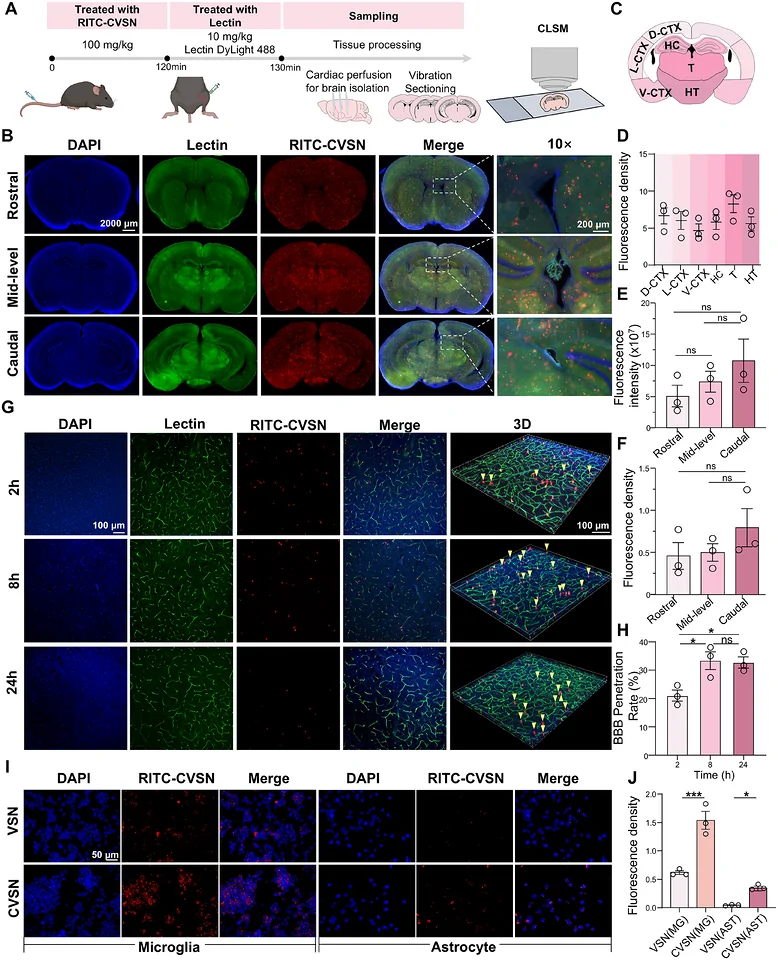
In vivo fate of CVSN in the brain. (A) Schematic of the experimental workflow for tissue distribution of CVSN in vivo. (B) Whole‐scan images of brain sections. Blue: DAPI‐stained nuclei; green: lectin‐stained vasculature; red: RITC‐CVSN. (C) Atlas of the hippocampal region (middle level). (D) Fluorescence intensity across different hippocampal subregions. Data are presented as mean ± SEM, *n* = 3, n refers to the number of biological replicates. (E) Overall fluorescence intensity of brain sections at different anatomical levels and (F) normalized fluorescence intensity per unit area. Data are presented as mean ± SEM, *n* = 3, n refers to the number of biological replicates. (G) CLSM images showing transvascular penetration of RITC‐labeled CVSN at 2 h. Blue: DAPI; green: Lectin; red: RITC‐CVSN. Yellow arrows indicate RITC‐CVSN penetrating the blood–brain barrier (BBB) into the parenchyma. (H) BBB permeability of RITC‐CVSN at different time points. Data are presented as mean ± SEM, *n* = 3, n refers to the number of biological replicates. *p < 0.05. (I) CLSM images of microglial uptake of RITC‐CVSN and (J) quantification of normalized fluorescence intensity per unit area. Blue: DAPI‐stained nuclei of microglia; red: RITC‐CVSN. Data are presented as mean ± SEM, *n* = 3, n refers to the number of biological replicates. *p < 0.05, **p < 0.01, ***p < 0.001, ****p < 0.0001. One‐way ANOVA post hoc Tukey's test was performed (D–F, H, J).

To further verify whether CVSN entered the parenchyma via the vasculature, cortical sections at three different time points were examined using CLSM. RITC‐CVSN exhibited two distinct patterns: partial adherence to the vascular wall and penetration into the brain parenchyma (Figure 3G), consistent with observations from Bio‐TEM. Quantitative analysis revealed a time‐dependent increase in BBB permeability, peaking at 8 h (approximately 35%) with no further significant change at 24 h (Figure 3H). These results indicate that CVSN crosses the BBB in a time‐dependent manner, thereby providing a mechanistic basis for its potential application in brain disease imaging, therapy, and targeted drug delivery. CLSM analysis of MSN and DVSN (Figure S9) similarly showed that a fraction of fluorescent nanoparticles from both groups penetrated the vasculature and entered the brain parenchyma. Notably, DVSN exhibited a significantly higher BBB penetration efficiency compared to MSN, further supporting the enhanced translocation capability conferred by the virus‐mimicking structure.

CLSM was employed to investigate the internalization efficiency of VSN and CVSN in microglia, astrocytes, and neurons. Imaging results demonstrated pronounced fluorescence signals of CVSN in the perinuclear region, which were markedly stronger than those of VSN. Quantitative analysis revealed that the fluorescence intensities of VSN and CVSN internalized by microglia were 0.620 and 1.539, respectively, while the corresponding intensities for astrocytes were 0.045 and 0.324 (Figure 3I,J). These findings indicate that immune cells effectively internalize nanoparticles, and that the chiral structure significantly enhances cellular uptake. Such internalization is favorable for modulating the immune‐inflammatory microenvironment upon drug loading. Furthermore, a fraction of nanoparticles was also internalized by neurons, with fluorescence intensities of 0.035 for VSN and 0.549 for CVSN (Figure S10). This suggests that drug‐loaded nanoparticles may indirectly mitigate the vicious cycle of inflammation, thereby attenuating neuronal damage. In addition, CX3CR1‐GFP transgenic mice were employed to further validate in vivo uptake of CVSN by microglia. CLSM revealed partial colocalization of CVSN with microglia (Figure S11), suggesting that microglia may participate in the clearance and homing processes of CVSN within the brain.

We performed immunofluorescence (IF) staining on brain sections from mice injected with RITC‐CVSN. The results revealed that CVSN exhibited strong colocalization with CD31, GFAP, and Iba1, whereas its colocalization with neurons was comparatively limited (Figure 4A–F). The colocalization with CD31 suggests improved drug distribution in the brain, reflecting the ability of CVSN to cross the blood–brain barrier. Meanwhile, the targeting of astrocytes (GFAP) and microglia (Iba1) highlights its potential to modulate neuroinflammatory responses and repair processes. Such multi‐targeted colocalization of the nanocarrier may contribute to comprehensive therapeutic outcomes.

**FIGURE 4:**
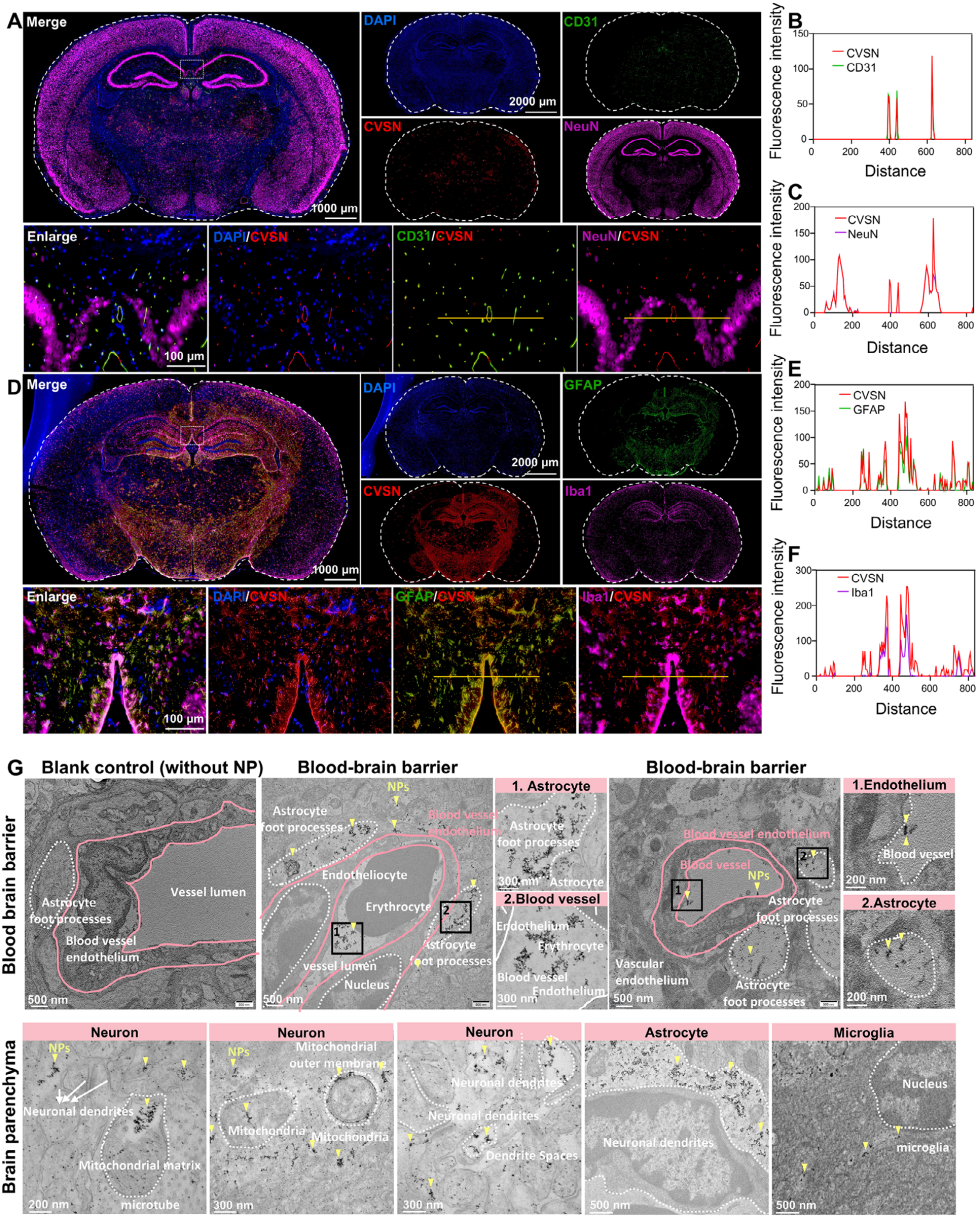
Colocalization of CVSN with different cell types. Immunofluorescence images obtained 2 h after intravenous injection of RITC‐CVSN show colocalization with cellular markers: CD31 and NeuN (A), and GFAP and Iba1 (D). Nuclei of mouse brain sections are stained with DAPI (blue); vascular endothelial cells/astrocytes are labeled in green; RITC‐CVSN is shown in red; and neurons/microglia are indicated in purple. (B,C, E,F) Line Scan Colocalization Analysis. (G) Bio‐TEM images of mouse brain at 2 h after intravenous injection of CVSN.

We further employed bio‐transmission electron microscopy (Bio‐TEM) to directly visualize CVSN translocation and distribution across the BBB. Abundant nanoparticles were observed in the BBB region (Figure 4G), primarily internalized by astrocytes and microglia, with some adhering to vascular walls. In non‐perfused brain sections, numerous particles were dispersed within vascular lumina. Moreover, nanoparticles were identified at both the inner and outer surfaces of neuronal mitochondria as well as in synaptic clefts. These findings suggest that the virus‐mimicking rough topology of CVSN enhances vascular retention, membrane penetration, and entry into brain parenchyma, where they are subsequently internalized by glial cells.

### 2.4 Biosafety of CVSN

To evaluate the potential toxicological effects of CVSN, in vitro cytotoxicity was first evaluated using the CCK‐8 assay. Within the concentration range of 0.01–50 µg mL^−1^, both VSN and CVSN exhibited no significant impact on cell viability, with survival rates consistently above 90% and no apparent dose‐dependent effects (Figure S12). Subsequently, the biosafety of CVSN was systematically examined both in vitro and in vivo.

In the hemolysis assay in vitro, which directly reflects the blood toxicity of nanomaterials, CVSN exhibited a slight concentration‐dependent increase in hemolysis but remained below 10% across 5–100 µg mL^−1^, meeting established safety thresholds for biomaterials (Figure S13A,B). Protein adsorption was evaluated using bovine serum albumin (BSA). The adsorption rate of CVSN toward BSA was less than 5% (Figure S13C), indicating low protein binding, that may help minimize the formation of protein‐nanomaterial complexes.

The mice were administered CVSN via tail vein injection and were observed for 14 days to assess the in vivo safety of CVSN (Figure S14A). Throughout the observation period, neither control nor CVSN‐treated participants displayed unusual lethargy, hypoactivity, anorexia, or convulsions. Body weight increased by 19.14% in the control group and 21.29% in the CVSN group (Figure S14B). On day 14, blood and vital organ specimens were collected for biochemical, hematological, and histopathological studies. No macroscopic deformities or organ index alterations were detected (Figure S14C). There was no significant difference between serum biochemical parameters in the CVSN group and saline group (Figure S14D), and all hematological indices were within normal reference ranges (Figure S15). In addition, hematoxylin‐eosin (HE) staining revealed no discernible tissue damage or pathological alterations (Figure S16). Together, the results show that CVSN is non‐toxic in vivo and in vitro, with remarkable biocompatibility and safety.

### 2.5 Transport Mechanism of CVSN Across the BBB

To elucidate the mechanism by which CVSN traverses the BBB, TEM was first employed to examine the BBB region. Internalized nanoparticles were observed within the cytoplasm of BBB endothelial cells (Figure 5A), suggesting that CVSN does not cross the BBB merely through passive leakage following barrier disruption, but rather via an active, endothelial cell‐mediated transcytosis process.

**FIGURE 5:**
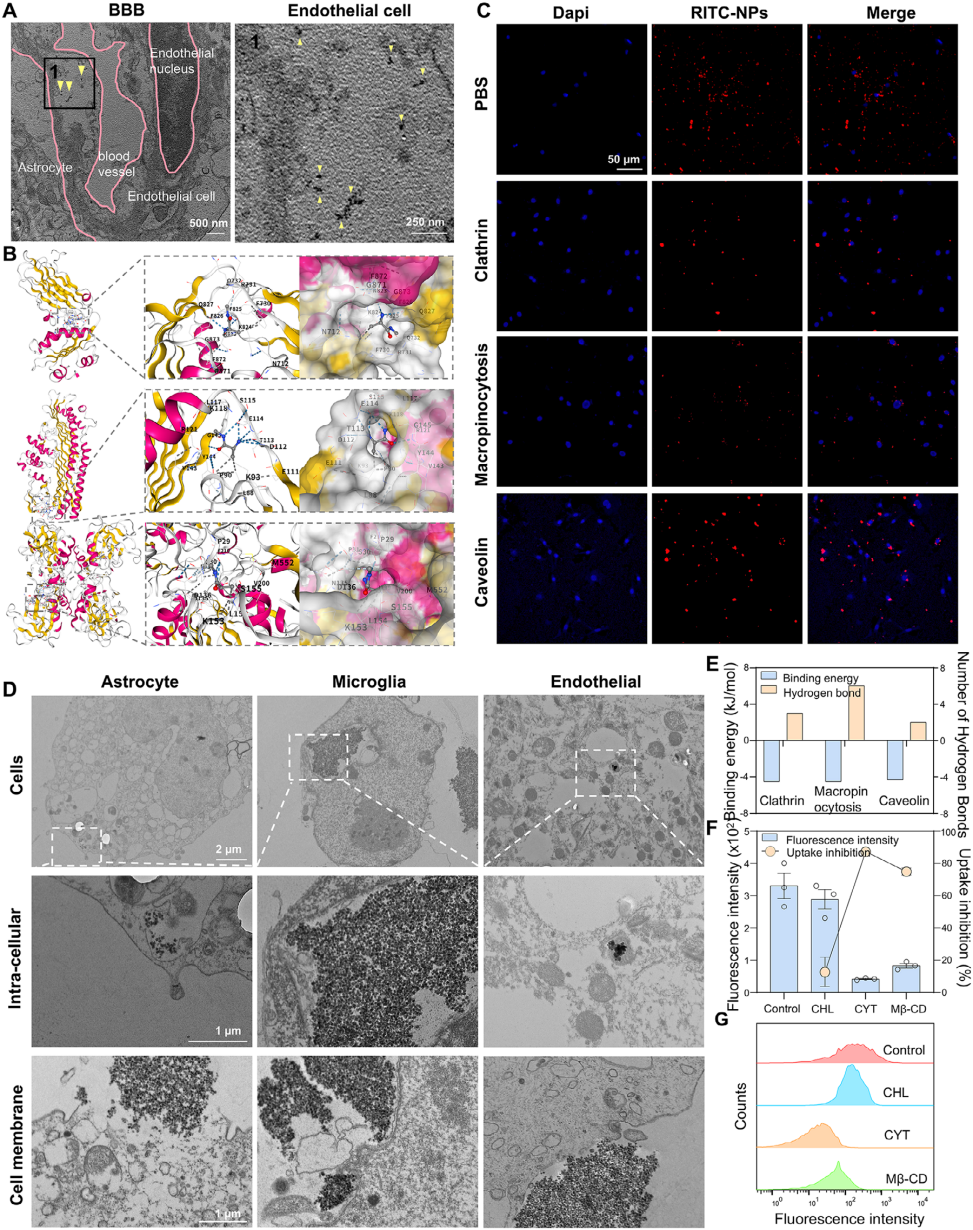
Mechanism of CVSN transport across the BBB. (A) Transmission electron microscopy images showing the uptake of CVSN by endothelial cells. (B) Molecular docking results of L‐ala with Clathrin‐, macropinocytosis‐, and Caveolin‐related molecules. (C) Uptake of RITC‐CVSN by endothelial cells under PBS control conditions and after inhibition of Clathrin, macropinocytosis, and Caveolin pathways. (D) Transmission electron microscopy images of CVSN uptake by microglia, astrocytes, and endothelial cells. (E) Statistical analysis of binding energies and numbers of hydrogen bonds in the molecular docking study. (F) Quantification of fluorescence intensity and inhibition rate of CVSN uptake by endothelial cells in the presence of inhibitors. Data are presented as mean ± SEM, *n* = 3, *n* refers to the number of biological replicates. (G) Flow cytometry analysis.

Given that L‐alanine is an exposed chiral ligand on the surface of CVSN and directly participates in the initial interaction and molecular recognition at the nano–bio interface, molecular docking analysis was performed using L‐alanine as the ligand against key proteins involved in endothelial uptake pathways. The results (Figure 5B,E) demonstrated favorable binding affinities between L‐alanine and clathrin‐associated proteins, macropinocytosis‐related proteins, and caveolae‐associated proteins, with binding energies of −4.5, −4.5, and −4.3 kcal mol^−1^, respectively, and hydrogen bond numbers of 3, 6, and 2. These findings suggest that the surface ligands of CVSN may participate in the recognition of endocytosis‐related proteins. In contrast, molecular docking results for DVSN (Figure S17) showed comparable binding energies (−4.2, −4.6, and −4.5 kcal mol^−1^), but significantly fewer hydrogen bonds (2, 3, and 2), indicating less stable molecular interactions. This suggests that CVSN may exhibit enhanced molecular recognition stability, thereby facilitating its interaction with endocytic structures on the cell membrane and subsequent cellular uptake.

To determine whether the chirality‐dependent nano‐bio interaction was specific to alanine, we further extended the surface modification strategy to two additional chiral amino acids, cysteine and arginine, generating VSN‐L‐Cys, VSN‐D‐Cys, VSN‐L‐Arg, and VSN‐D‐Arg. Molecular docking showed that inversion of ligand chirality altered the interaction modes with proteins associated with clathrin‐mediated endocytosis, macropinocytosis, and caveolae‐mediated endocytosis. For cysteine, L‐cysteine generally exhibited more favorable binding energies than D‐cysteine, whereas differences in hydrogen‐bond numbers varied among the three endocytic pathways (Figure S18). For arginine, the L‐ and D‐enantiomers displayed broadly comparable binding energies but distinct hydrogen‐bonding patterns across the tested proteins (Figure S19). These findings indicate that stereochemical configuration modulates molecular recognition at the nano‐bio interface, while the detailed interaction pattern is also influenced by the chemical properties of the amino‐acid side chain.

To further determine whether these stereochemistry‐dependent molecular interactions translated into differences at the cellular and in vivo levels, endothelial uptake and organ biodistribution were evaluated. Time‐dependent ex vivo fluorescence imaging following intravenous administration revealed predominant hepatic accumulation for all formulations, consistent with systemic clearance of silica‐based nanoparticles. Notably, VSN‐L‐Cys and VSN‐L‐Arg exhibited higher overall fluorescence signals in the brain than their corresponding D‐enantiomer‐functionalized counterparts during the observation period (Figures S20 and S21). Consistently, CLSM analysis of bEnd.3 endothelial cells showed stronger intracellular RITC fluorescence in the VSN‐L‐Cys and VSN‐L‐Arg groups than in the corresponding VSN‐D‐Cys and VSN‐D‐Arg groups, respectively (Figure S22). Together, these findings demonstrate that the chirality‐dependent enhancement of endothelial interaction and brain accumulation is not unique to alanine and can be reproduced with at least two additional chiral amino‐acid ligands. However, the magnitude and molecular basis of this stereoselective effect vary with ligand structure, suggesting that surface chirality acts in concert with side‐chain chemistry rather than through a single universal molecular interaction.

We next investigated whether the level of L‐alanine functionalization influenced cellular recognition and uptake by brain endothelial cells. Using the CVSN data under PBS conditions from Figure 5C as the CVSN (1L‐Ala) group, we compared its intracellular RITC fluorescence with that of unmodified VSN and the other L‐alanine‐functionalized formulations (Figure S23). Confocal laser scanning microscopy (CLSM) analysis showed that CVSN (1L‐Ala) exhibited significantly stronger intracellular RITC fluorescence than unmodified VSN, CVSN (1/2L‐Ala), and CVSN (2L‐Ala). Importantly, both decreasing the L‐alanine feed to 0.5‐fold and increasing it to 2‐fold significantly reduced cellular fluorescence compared with CVSN(1 × L‐Ala). Thus, increasing the amount of L‐alanine did not monotonically enhance cellular uptake. Instead, the standard CVSN formulation exhibited the highest uptake efficiency among the tested formulations. These results demonstrate a functionalization‐level‐dependent effect of L‐alanine on nano‐bio recognition and support the existence of an optimal range of surface ligand presentation for efficient endothelial internalization.

Subsequently, endothelial cells were pretreated with inhibitors of different endocytic pathways and co‐incubated with RITC‐labeled CVSN, followed by CLSM observation (Figure 5C). The results revealed that chlorpromazine (CHL) exerted a relatively weak inhibitory effect, whereas cytochalasin D (CYT) and methyl‐β‐cyclodextrin (Mβ‐CD) markedly reduced intracellular red fluorescence signals. Quantitative analysis (Figure 5F) showed inhibition rates of 12.73% for CHL, compared to 87.31% and 74.90% for CYT and Mβ‐CD, respectively. These findings indicate that CVSN is primarily internalized via macropinocytosis and caveolae‐mediated endocytosis.

The control material DVSN exhibited a similar trend (Figure S24), with relatively weak inhibition by CHL and significant reduction in uptake upon CYT and Mβ‐CD treatment, with inhibition rates of 29.3%, 79.98%, and 71.57%, respectively. This further supports that chiral‐modified nanostructures predominantly rely on macropinocytosis and caveolae‐mediated pathways for cellular entry. In contrast, MSN displayed a distinct uptake profile (Figure S25), where CHL and CYT exerted weaker effects, while Mβ‐CD caused more pronounced inhibition, with inhibition rates of 68.62%, 81.23%, and 27.39%, respectively, suggesting that MSN uptake is more dependent on clathrin‐mediated endocytosis. Collectively, these results indicate that chiral modification and virus‐like surface architecture can alter nanoparticle internalization pathways, shifting from classical clathrin‐mediated endocytosis toward macropinocytosis and caveolae‐mediated uptake.

Flow cytometry analysis (Figure 5G) was consistent with the CLSM findings, further confirming that CVSN predominantly enters endothelial cells via macropinocytosis and caveolae‐mediated pathways, with limited contribution from clathrin‐mediated endocytosis. Additionally, TEM observations (Figure 5D) revealed that nanoparticles were encapsulated within membrane‐bound vesicular structures in microglia, astrocytes, and endothelial cells, indicating that endocytosis is the primary mode of cellular uptake across different brain‐associated cell types. Similar TEM observations were obtained for MSN (Figure S26), suggesting that mesoporous silica‐based nanocarriers can also enter cells via endocytosis, although their specific internalization pathways are strongly influenced by surface structure and functional modifications.

In summary, the efficient transport of CVSN across the BBB and its therapeutic delivery in bacterial meningitis rely on a multifactorial synergistic mechanism, including: (1) **Passive targeting under pathological conditions and structural advantages**: During bacterial meningitis, inflammatory responses disrupt BBB integrity, downregulate tight junction proteins, and significantly increase permeability, facilitating the penetration of small‐sized nanoparticles into brain tissue. (2) **Enhanced active uptake via chiral recognition**: L‐alanine modification confers more stable molecular interactions (higher hydrogen bond numbers), thereby improving cellular uptake efficiency. In contrast, DVSN and MSN exhibit weaker or less specific interactions, highlighting the critical role of chirality in BBB translocation. (3) **Multivalent anchoring effect**: The virus‐like rough surface provides multiple contact points, enhancing membrane adhesion and local accumulation, and promoting caveolae‐ and lipid raft‐associated endocytosis. (4) **Synergistic endocytosis and transcytosis**: CVSN is predominantly internalized by endothelial cells via macropinocytosis and caveolae‐mediated pathways, followed by transcytosis across the BBB, and is subsequently taken up by microglia and astrocytes.

Together, CVSN achieves efficient BBB penetration and targeted brain delivery through the synergistic interplay of pathological barrier modulation, structural features, chiral regulation, and interfacial interactions.

### 2.6 Construction of the Drug Delivery System

To achieve CVSN‐mediated treatment of meningitis, CPZ was loaded into the mesopores of CVSN via a solvent evaporation method to obtain CVSN@CPZ (Figure 6A). TEM characterization revealed no significant changes in morphology or particle size following drug loading (Figure 6B). In nitrogen adsorption/desorption analysis, compared with CVSN, CVSN@CPZ displayed improved curve overlap, disappearance of the H1‐type hysteresis loop, and a typical type II isotherm profile, consistent with a nonporous structure (Figure 6C), confirming mesopore occupation. The BJH pore distribution shifted from a pronounced peak in CVSN to a smooth curve in CVSN@CPZ (Figure 6D), with no distinct pore distribution observed. After drug loading, both BET surface area and pore volume were significantly reduced, with complete loss of micropore distribution (Table S1). Infrared spectra showed that CPZ exhibits a characteristic absorption peak at ∼1500 cm^−1^; however, the spectrum of CVSN@CPZ closely resembled that of CVSN, with most CPZ peaks disappearing (Figure 6E), suggesting successful encapsulation and confinement of CPZ within the mesopores, where interactions with the carrier altered its spectral features. XRD analysis showed that CVSN exhibited an amorphous structure, while CPZ displayed sharp crystalline peaks between 5° and 50°, indicative of crystalline characteristics. In contrast, CVSN@CPZ showed no distinct crystalline phase (Figure 6F), implying that CPZ transitioned from a crystalline to an amorphous state upon encapsulation. Surface charge remained stable (Figure 6G), indicating favorable colloidal stability in physiological environments.

**FIGURE 6:**
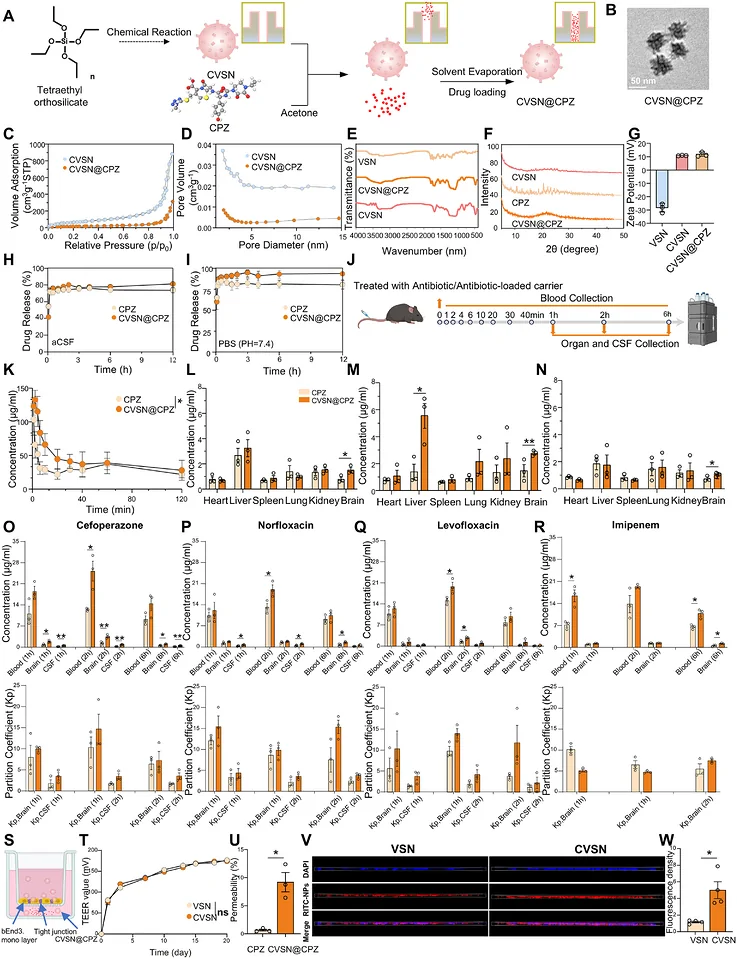
Properties of drug‐loaded carriers and pharmacokinetics. (A) Schematic illustration of drug‐loaded carrier synthesis. (B) TEM image of CVSN@CPZ. (C) Nitrogen adsorption/desorption isotherms. (D) Pore size distribution of CVSN@CPZ. (E) FTIR spectra. (F) XRD patterns. (G) Zeta potential. Data are presented as mean ± SEM, *n* = 3, n refers to the number of biological replicates. (H) Drug release profiles of CPZ and CVSN@CPZ in aCSF and (I) in PBS (pH = 7.4). Data are presented as mean ± SEM, *n* = 3, n refers to the number of biological replicates. (J) Schematic of the pharmacokinetic study design based on HPLC quantification. (K) Plasma concentrations of CPZ at different time points after intravenous injection of CPZ and CVSN@CPZ. Data are presented as mean ± SEM, *n* = 3, n refers to the number of biological replicates. (L‐N) Tissue distribution of CPZ in major organs at (L) 1 h, (M) 2 h, and (N) 6 h after intravenous injection of CPZ and CVSN@CPZ. Time‐dependent drug concentrations in plasma, major organs, brain, and CSF, as well as brain‐to‐plasma and CSF‐to‐plasma ratios for (O) CPZ and CVSN@CPZ, (P) NOR and CVSN@NOR, (Q) LFX and CVSN@LFX, and (R) IPM and CVSN@IPM. Data are presented as mean ± SEM, *n* = 3, n refers to the number of biological replicates. (S) Schematic illustration of the bEnd3‐based transwell model mimicking the BBB in vitro. (T) Changes in transmembrane potential of the monolayer at different time points. *n* = 3, n refers to the number of biological replicates. (U) CPZ and CVSN@CPZ were added to the upper chamber of a monolayer membrane, and the solution collected from the lower chamber after 2 h was analyzed by HPLC to quantify CPZ content, *n* = 3, n refers to the number of biological replicates. (V) RITC‐CVSN was added to the upper chamber of a monolayer membrane, and the solution collected from the lower chamber after 2 h was subjected to CLSM Z‐stack imaging. (W) Fluorescence intensity per unit area was quantified using ImageJ. *n* = 3, n refers to the number of biological replicates. *p < 0.05, **p < 0.01, ***p < 0.001, ****p < 0.0001. Two‐way ANOVA with Bonferroni's multiple comparisons test was performed (K, T). Two‐sided unpaired t‐test was performed (L–R, U, W).

In vitro release studies showed that in simulated body fluid (pH 7.4 PBS), free CPZ released ∼80% within 15 min and plateaued at ∼85% after 12 h. In contrast, CVSN@CPZ exhibited an initial release of ∼87% within 15 min, followed by a slower release, reaching a plateau at ∼100% within 2 h (Figure 6I). In aCSF, CVSN@CPZ reached a plateau within 1 h, with slightly higher cumulative release compared to free CPZ (Figure 6H). These results indicate that CVSN enhanced both the release rate and extent of CPZ in simulated body fluid and aCSF.

For in vivo pharmacokinetics and biodistribution studies, high‐performance liquid chromatography (HPLC) was used to quantify plasma concentrations of CVSN@CPZ over time, as well as its distribution in major organs and CSF (Figure 6J). Following administration, plasma concentrations of both CVSN@CPZ and CPZ peaked rapidly, with Cmax values of 132.93 and 103.40 µg mL^−1^, respectively, followed by multiphasic declines. The concentration‐time curves differed significantly between groups (Figure 6K), indicating that CVSN@CPZ markedly improved the bioavailability of CPZ. Both formulations were detectable and widely distributed across tested tissues. At 1, 2, and 6 h, brain concentrations in the CVSN@CPZ group were significantly higher than those in the CPZ group, with plasma concentrations also differing notably at 2 h. Over time, these differences gradually diminished (Figure 6L–N).

### 2.7 Intravenous Administration and Biodistribution of Antibiotic‐Loaded Nanoparticles

Based on our previous findings, CVSN has been established as an efficient nanocarrier that markedly enhances the bioavailability of native antibiotics. Universality is critical criterion for evaluating the translational potential and quality of a delivery platform. To assess whether CVSN possesses broad applicability for different antibiotics, we selected three representative drugs with distinct physicochemical and pharmacological properties: NOR, LFX, and IPM. These antibiotics differ in class, molecular weight, solubility, and chirality (Table S2), as well as in antibacterial spectra and BBB permeability (Table S3). This design enables comprehensive evaluation of the generalizability of the CVSN‐based delivery system across multiple dimensions. Using the same loading method as for CPZ, NOR, LFX, and IPM were incorporated into the mesoporous structure of CVSN. Standard calibration curves for each antibiotic were established using UV spectrophotometry (Figure S27). The calculated drug‐loading capacities of CVSN@CPZ, CVSN@NOR, CVSN@LFX, and CVSN@IPM were 41.29 ± 1.25%, 34.89 ± 0.65%, 33.18 ± 1.08%, and 25.67 ± 1.01%, respectively, with corresponding encapsulation efficiencies of 82.57 ± 2.50%, 69.77 ± 1.31%, 66.36 ± 2.16%, and 51.33 ± 2.02% (Figure S28). These results demonstrate that CVSN can efficiently load a broad range of antibiotics, and its high encapsulation efficiency facilitates improved drug utilization. Furthermore, release experiments in vitro in simulated body fluid (pH 7.4 PBS) revealed that antibiotic‐loaded CVSN exhibited higher cumulative release and a prolonged release plateau, indicating a sustained‐release property (Figure S29).

To evaluate the universality of BBB penetration for different antibiotic‐loaded nanoparticles, HPLC conditions were optimized for the quantification of each antibiotic (Table S4 and Figure S30). The ability of drugs to enter CSF is a critical determinant of their potential to access the ventricular system and exert therapeutic effects through CSF circulation in meningitis. To ensure the reliability of CSF sampling, we used glass microelectrodes to collect CSF from the cisterna magna (Figure S31). Antibiotic concentrations in plasma, brain tissue, and CSF were subsequently determined at three time points. The results revealed notable differences in distribution among the antibiotics following CVSN loading. Importantly, except for imipenem, the other three groups demonstrated relatively high antibiotic concentrations in CSF. Further analysis revealed that the ratio of brain‐to‐plasma concentrations (Kp, Brain) directly reflected BBB penetration and accumulation in brain parenchyma, whereas the CSF‐to‐plasma ratio (Kp, CSF) represented the ability of drugs to enter CSF. Notably, both Kp (Brain) and Kp (CSF) values were significantly elevated for all four antibiotics following CVSN loading (Figure 6O–R), suggesting that CVSN markedly enhances BBB penetration and improves the efficiency of drug entry into the CSF. In contrast, no significant differences were observed in either Kp (Brain) or Kp (CSF) between the VSN@CPZ group and the control group (Figure S32), indicating that, compared with VSN, CVSN more effectively enhances BBB penetration and promotes drug delivery into the CSF.

Considering its broad antibacterial spectrum, intrinsically poor BBB penetration, high drug‐loading capacity, favorable brain distribution upon CVSN encapsulation, and excellent in vivo biodistribution, CPZ was ultimately selected as the primary candidate for subsequent investigations. To mimic the BBB, we established a monolayer membrane model using Bends3 cells to evaluate the permeability of CPZ and CVSN (Figure 6S). The integrity of the monolayer was first assessed by measuring the transendothelial electrical resistance (TEER). The results showed that the resistance stabilized at approximately 170 mV (>150 mV) after 15–20 days (Figure 6T), indicating the successful formation of tight junctions and validating the model for BBB permeability studies. The permeability assay revealed that CPZ alone exhibited poor penetration, with an efficiency of only 0.65%. In contrast, CVSN@CPZ markedly enhanced CPZ penetration, achieving an efficiency of 9.18% (Figure 6U). As a third‐generation cephalosporin antibiotic, CPZ demonstrates limited ability to cross an intact BBB, whereas CVSN functions as an effective “vehicle” to facilitate its transport. Considering the compromised BBB integrity in meningitis patients, the penetration efficiency of CVSN@CPZ would likely be further improved in pathological conditions. Moreover, we also evaluated the intrinsic BBB permeability of the carrier itself. Nanoparticle penetration assays demonstrated that CVSN, compared with VSN, significantly enhanced BBB penetration and was more efficiently internalized by endothelial cells (Figure 6V,W).

### 2.8 Antibacterial Activity In Vitro

We first performed a preliminary screening of CPZ against common clinical meningitis pathogens. Heatmap analysis revealed that CPZ exhibited strong antibacterial activity against *Escherichia coli* (ETEC) and *Staphylococcus aureus* (SAU), with inhibition rates exceeding 90% (Figure 7A). Therefore, we further conducted susceptibility testing of free CPZ and CVSN@CPZ against ETEC and SAU. The minimum inhibitory concentration (MIC) of CPZ was 16 µg mL^−1^ for ETEC (Figure 7B) and 512 µg mL^−1^ for SAU (Figure 7C), indicating greater tolerance of SAU to CPZ. In contrast, the MICs of CVSN@CPZ decreased to 8 µg mL^−1^ for ETEC (Figure 7D) and 256 µg mL^−1^ for SAU (Figure 7E). This improvement can be ascribed to the high drug bioavailability, mediated by effective drug release, and nanoparticle‐mediated attack on bacterial surfaces, increased local drug concentrations, and enhanced antibacterial effects (Figure 7F).

**FIGURE 7:**
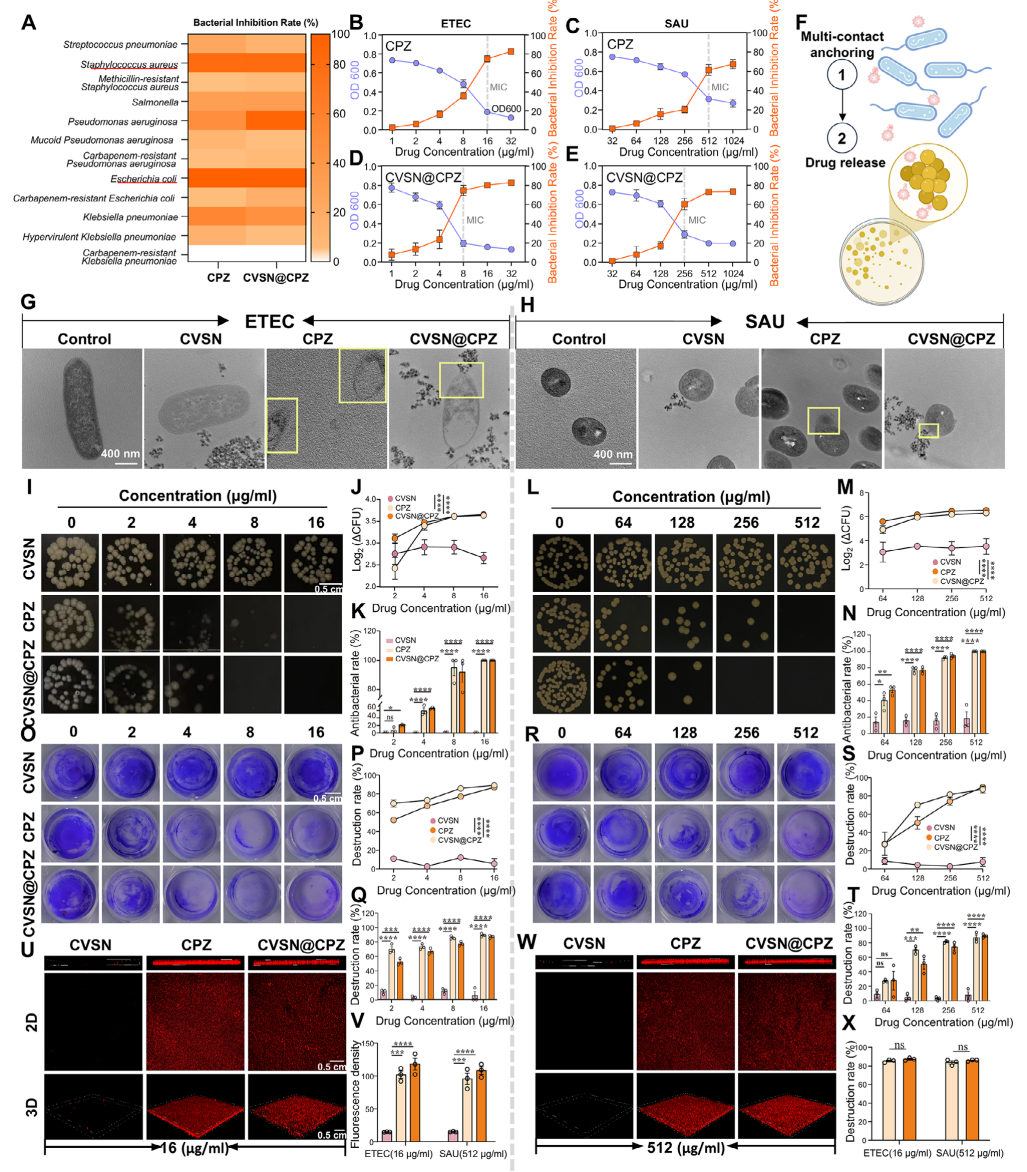
Antibacterial effects of CVSN before and after drug loading against *Escherichia coli* and *Staphylococcus aureus in vitro*. (A) Heatmap of CPZ antibacterial activity screening. (B) Antibacterial assay statistics of ETEC and (C) SAU after co‐incubation with CPZ solution. The concentration of the drug‐loaded carrier was calculated based on the drug‐loading rate to reflect the equivalent concentration of raw CPZ. Antibacterial assay statistics of (D) ETEC and (E) SAU after co‐incubation with CVSN@CPZ solution. Data are presented as mean ± SEM, *n* = 3, n refers to the number of biological replicates. (F) Schematic illustration of the antibacterial mechanism of CVSN@CPZ. Illustration created with BioRender. TEM images showing the inhibitory effect of CVSN@CPZ on (G) ETEC and (H) SAU after co‐incubation. Colony growth images of (I) ETEC and (L) SAU treated with different concentrations of CVSN, CVSN@CPZ, and CPZ. Quantification of colony reduction in (J) ETEC and (M) SAU treated with different concentrations of CVSN, CVSN@CPZ, and CPZ. Statistical analysis of inhibition rates of (K) ETEC and (N) SAU under the same treatments. Data are presented as mean ± SEM, *n* = 3, n refers to the number of biological replicates. Crystal violet staining images of (O) ETEC and (R) SAU biofilm inhibition treated with different concentrations of CVSN, CVSN@CPZ, and CPZ. Line‐plot statistics of biofilm disruption rates in (P) ETEC and (S) SAU under the same treatments. Data are presented as mean ± SEM, *n* = 3, n refers to the number of biological replicates. Statistical analysis of inhibition rates of (Q) ETEC and (T) SAU under the same treatments. Data are presented as mean ± SEM, *n* = 3, n refers to the number of biological replicates. Dead staining images showing antibacterial effects of CVSN, CVSN@CPZ, and CPZ against (U) ETEC and (W) SAU. Statistical analysis of (V) fluorescence intensity per unit area and (X) inhibition rates of ETEC and SAU after live/dead staining. Data are presented as mean ± SEM, *n* = 3, n refers to the number of biological replicates. *p < 0.05, **p < 0.01, ***p < 0.001, ****p < 0.0001. Two‐way ANOVA with Bonferroni's multiple comparisons test was performed (J, M, P, S). One‐way ANOVA post hoc Tukey's test was performed (K, N, Q, T, V). Two‐sided unpaired t‐test was performed (X).

Bio‐TEM analysis confirmed that both bacterial strains showed disrupted cell wall structures following post‐incubation with CPZ or CVSN@CPZ with cells in the PBS control group maintaining the morphology of the cells intact (Figure 7G,H). This would imply that exposure to CVSN facilitates the better binding of CPZ to bacterial cells leading to selective release and potentiation of bactericidal activity. In the VSN@CPZ‐treated group (Figure S33), *Escherichia coli* exhibited pronounced cellular damage, whereas no obvious structural disruption was observed in *Staphylococcus aureus*. In colony growth, colony numbers reduced gradually with increases in drug use in CPZ and CVSN@CPZ (Figure 7I,L). Complete inhibition of colony growth was however detected at ETEC up to 16, 8 and 512, and SAU for CPZ and CVSN@CPZ, 16 and 8 µg mL^−1^ for ETEC, and 512 and 256 µg mL^−1^ for SAU, respectively. Neither CVSN, individually, produced an obvious antibacterial effect (Figures 7J‐K, 7M‐N) and was consistent with the susceptibility values from others in the case. Compared with CVSN@CPZ, VSN@CPZ showed significant antibacterial effects against ETEC at 16 µg mL^−1^ (Figure S34A) and against SAU at 512 µg mL^−1^ (Figure S34F), but did not achieve complete bacterial elimination (Figure S34B–E). These results further demonstrate the superior bactericidal performance and higher treatment precision of CVSN@CPZ for antibacterial therapy.

After colonization in the meningeal cavity, bacteria secrete extracellular polysaccharides and proteins to form biofilms, thereby enhancing resistance to host immune clearance and antimicrobial agents [33]. To evaluate the inhibitory effects of drugs and drug‐loaded systems on bacterial biofilm formation, we performed visual analyses using crystal violet staining. Increasing drug concentrations led to a progressive disruption of biofilms by both CPZ and CVSN@CPZ, with more pronounced effects observed in the ETEC group; in contrast, the CVSN control group exhibited no obvious impact (Figure 7O–T). Since bacterial growth assays and crystal violet staining cannot distinguish between live and dead bacteria, we further applied propidium iodide (PI) staining to detect bacterial death (Figure 7U,W). The results revealed that under conditions of 16 µg mL^−1^ for ETEC and 512 µg mL^−1^ for SAU, the numbers of dead bacteria in the CPZ and CVSN@CPZ treatment groups were significantly higher than those in the control groups (Figure 7V,X). The same results were observed at lower concentrations (8 µg mL^−1^ for ETEC and 128 µg mL^−1^ for SAU) (Figure S35). On the other hand, applying CPZ into CVSN showed an in vitro increased antibacterial activity of CPZ against both ETEC and SAU significantly while reducing MIC values and had much higher damaging effects on bacterial cell walls and biofilms to exhibit a much larger percentage of bacterial death.

Collectively, these findings collectively show that CVSN itself has no antibacterial activity and the antibacterial behavior of CPZ against both ETEC and SAU is dramatically increased upon loading with CVSN. In particular, CVSN@CPZ not only decreased MIC values but also increased disruption of the bacterial cell walls and biofilms and therefore also results in a higher proportion of bacterial death.

### 2.9 Therapeutic Efficacy of CVSN@CPZ in Bacterial Meningitis

Given the excellent in vitro antibacterial performance of CVSN@CPZ, we further established murine meningitis models infected with ETEC and SAU and performed drug treatments to systematically evaluate its therapeutic efficacy in vivo (Figure 8A and Figure 9A). Within five days after model induction, sham‐operated mice exhibited normal behavior, whereas model mice developed severe clinical symptoms, including hunching, tremors, and ataxia. Following CVSN@CPZ treatment, these symptoms were markedly alleviated. In bacterial culture assays (Figure 8B and Figure 9B), no colonies were detected in the CSF of sham‐operated mice, confirming a sterile baseline environment in the absence of bacterial inoculation. In contrast, substantial colony formation was observed in the untreated model group, verifying successful construction of the meningitis model. Colony numbers were moderately reduced in the CPZ group, while the CVSN@CPZ group exhibited a significant reduction. Quantitative analysis showed that in the ETEC model, the inhibition rate increased from 96.69% with CPZ to 97.64% with CVSN@CPZ; in the SAU model, the inhibition rate improved from 79.66% with CPZ to 94.75% with CVSN@CPZ (Figure 8C and Figure 9C), highlighting the superior antibacterial efficacy of the nanocarrier system over free drug.

**FIGURE 8:**
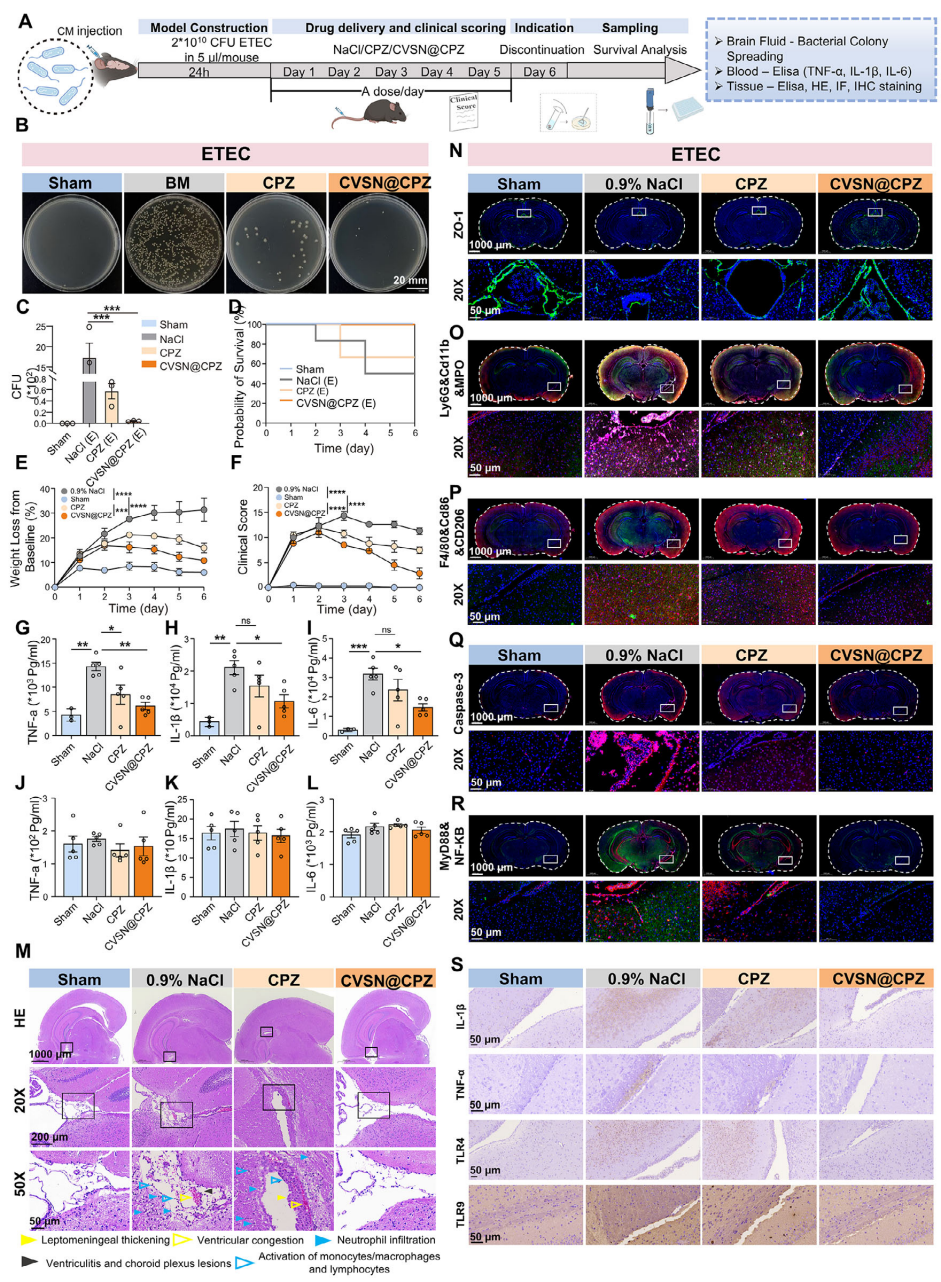
Therapeutic Evaluation and Molecular Mechanisms of CVSN@CPZ in ETEC—Induced BM. (A) Experimental design, therapeutic strategy of CVSN@CPZ, and evaluation methods in mouse models of ETEC—Induced BM. The injection dose of CPZ is 2 mg kg^−1^, and the injection dose of CVSN@CPZ is 4.84 mg kg^−1^ (calculated based on the drug loading rate). (B) Representative CSF bacterial colony growth images from ETEC groups. (C) Quantification of colony‐forming units (CFUs) in CSF from the ETEC group (*n* = 3, n refers to the number of biological replicates.). Data are presented as mean ± SEM. (D) Survival curves of the ETEC group (*n* = 6, n refers to the number of biological replicates.). (E) Body weight changes in the ETEC group (*n* = 6, *n* refers to the number of biological replicates.). Data are presented as mean ± SEM. (F) Clinical scores of the ETEC group (*n* = 6, n refers to the number of biological replicates.). Data are presented as mean ± SEM. Cytokine levels in the brain tissues of ETEC‐infected mice before and after treatment: (G) TNF‐α, (H) IL‐1β, and (I) IL‐6 (*n* = 3‐5, n refers to the number of biological replicates.). Data are presented as mean ± SEM. Cytokine levels in blood samples of ETEC‐infected mice before and after treatment: (J) TNF‐α, (K) IL‐1β, and (L) IL‐6 (*n* = 5, n refers to the number of biological replicates.). Data are presented as mean ± SEM. No significant difference observed. (M) HE staining results of brain sections from mice with meningitis in ETEC group. (N) ZO‐1 staining. Blue: cell nuclei stained with DAPI; Green: neutrophils. (O) Ly6G, Cd11b, and MPO staining. Blue: cell nuclei stained with DAPI; Red: myeloid cells; Purple: myeloperoxidase (MPO) protein. (P) F4/80, Cd86, and CD206 staining. Blue: cell nuclei stained with DAPI; Green: M1 macrophages; Red: M2 macrophages. (Q) Caspase‐3 staining. Blue: cell nuclei stained with DAPI; Red: apoptotic cells. (R) NF‐κB and MyD88 staining. Blue: cell nuclei stained with DAPI; Red: NF‐κB; Green: MyD88. (S) IHC staining of IL‐1β, TNF‐α, TLR4, and TLR9 before and after CVSN@CPZ administration. Source data are provided in the source data file. *p < 0.05, **p < 0.01, ***p < 0.001, ****p < 0.0001. Two‐way ANOVA with Bonferroni's multiple comparisons test was performed (E,F). One‐way ANOVA post hoc Tukey's test was performed (D, G‐L, Figures S40–S43).

**FIGURE 9:**
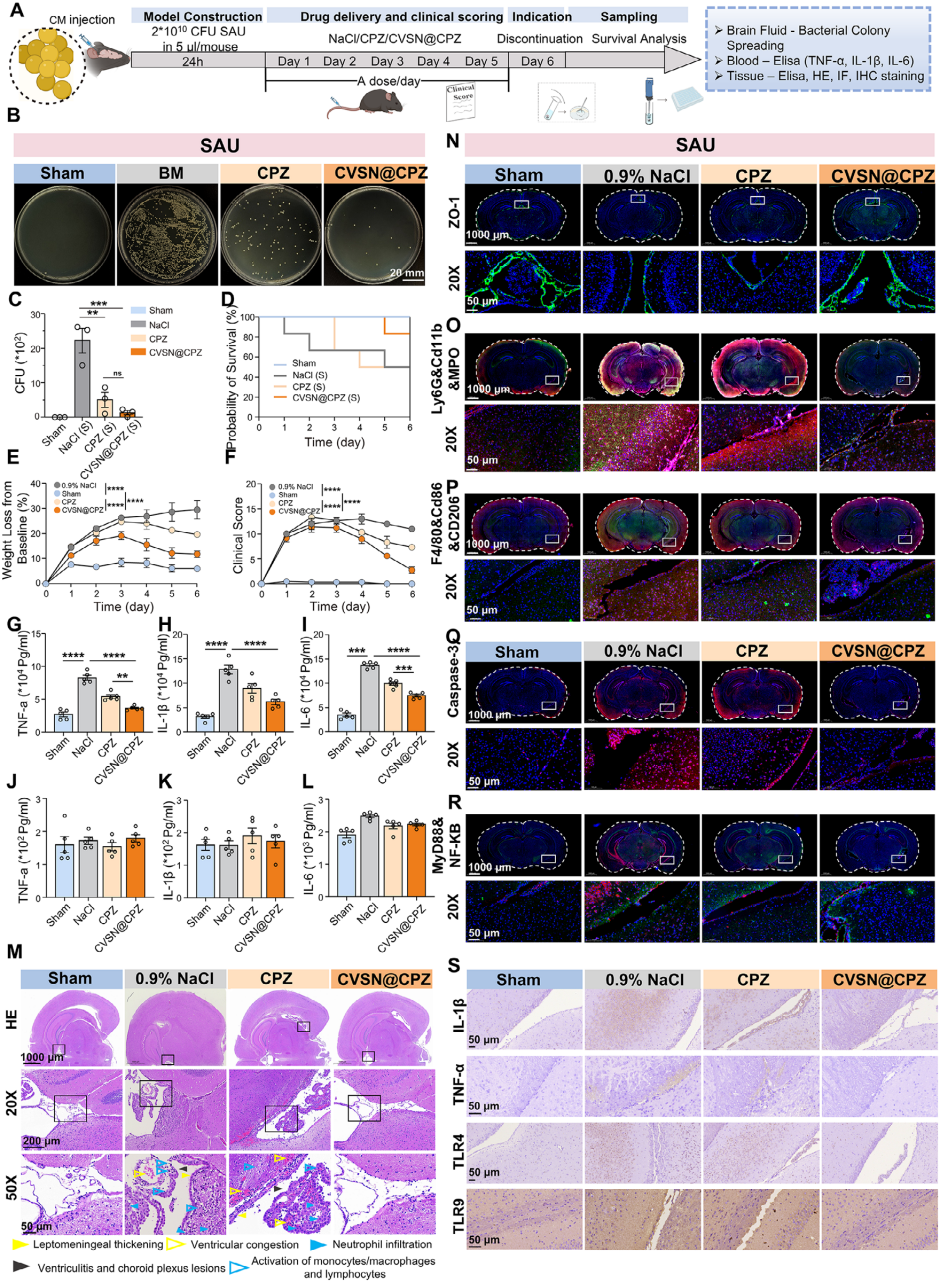
Therapeutic Evaluation and Molecular Mechanisms of CVSN@CPZ in SAU—Induced BM. (A) Experimental design, therapeutic strategy of CVSN@CPZ, and evaluation methods in mouse models of SAU—Induced BM. The injection dose of CPZ is 2 mg kg^−1^, and the injection dose of CVSN@CPZ is 4.84 mg kg^−1^ (calculated based on the drug loading rate) (B) Representative CSF bacterial colony growth images from SAU groups. (C) Quantification of colony‐forming units (CFUs) in CSF from the SAU group (*n* = 3, n refers to the number of biological replicates.). Data are presented as mean ± SEM. (D) Survival curves of the SAU group (*n* = 6, n refers to the number of biological replicates.). (E) Body weight changes in the SAU group (*n* = 6, n refers to the number of biological replicates.). Data are presented as mean ± SEM. (F) Clinical scores of the SAU group (*n* = 6, n refers to the number of biological replicates.). Data are presented as mean ± SEM. Cytokine levels in the brain tissues of SAU‐infected mice before and after treatment: (G) TNF‐α, (H) IL‐1β, and (I) IL‐6 (*n* = 5, n refers to the number of biological replicates.). Data are presented as mean ± SEM. Cytokine levels in blood samples of SAU‐infected mice before and after treatment: (J) TNF‐α, (K) IL‐1β, and (L) IL‐6 (*n* = 5, n refers to the number of biological replicates.). Data are presented as mean ± SEM. No significant difference observed. (M) HE staining results of brain sections from mice with meningitis in SAU group. (N) ZO‐1 staining. Blue: cell nuclei stained with DAPI; Green: neutrophils. (O) Ly6G, Cd11b, and MPO staining. Blue: cell nuclei stained with DAPI; Red: myeloid cells; Purple: myeloperoxidase (MPO) protein. (P) F4/80, Cd86, and CD206 staining. Blue: cell nuclei stained with DAPI; Green: M1 macrophages; Red: M2 macrophages. (Q) Caspase‐3 staining. Blue: cell nuclei stained with DAPI; Red: apoptotic cells. (R) NF‐κB and MyD88 staining. Blue: cell nuclei stained with DAPI; Red: NF‐κB; Green: MyD88. (S) IHC staining of IL‐1β, TNF‐α, TLR4, and TLR9 before and after CVSN@CPZ administration. Source data are provided in the source data file. *p < 0.05, **p < 0.01, ***p < 0.001, ****p < 0.0001. Two‐way ANOVA with Bonferroni's multiple comparisons test was performed (E,F). One‐way ANOVA post hoc Tukey's test was performed (D, G–L, Figures S40–S43). The Sham group is shared with Figure 8. The same representative Sham image is shown in Figure 8 and Figure 9 for comparison.

Regarding survival and clinical outcomes, untreated mice in the ETEC group displayed a survival rate of only 50% within six days, progressive increases of clinical scores, and up to 31.31% body weight loss, indicative of disease progression. CPZ treatment modestly improved survival to 66.67% and partially reversed weight loss by day 5. Remarkably, CVSN@CPZ treatment resulted in 100% survival, minimal weight loss, and near‐normal clinical scores (2.83) (Figure 8D–F). In the SAU model, untreated mice also had 50% survival within six days, accompanied by body weight loss of 29.49% and clinical scores reaching as high as 13. CPZ treatment did not improve survival, which remained at 50%, with only slight symptomatic relief. In contrast, CVSN@CPZ treatment significantly improved survival to 83.33%, mitigated body weight loss, and markedly alleviated clinical symptoms (Figure 9D–F).

Analysis of inflammatory cytokines further validated the therapeutic potential of CVSN@CPZ. In both ETEC and SAU models, untreated brain tissues showed significantly elevated TNF‐α, IL‐1β, and IL‐6 levels, indicating severe neuroinflammation. CPZ treatment reduced TNF‐α but had little effect on IL‐1β or IL‐6. By comparison, CVSN@CPZ significantly reduced all three cytokines (Figure 8G–I and Figure 9G–I), demonstrating that its benefits extend beyond antibacterial activity to effective suppression of inflammation. Notably, cytokine levels in blood samples showed no significant differences among groups (Figure 8J–L and Figure 9G–L), suggesting that the model specifically induced localized neuroinflammation without systemic inflammatory interference.

Histopathologic analysis of brain tissue showed characteristic BM pathology in the model group such as thickening of meningeal surfaces, neutrophil infiltration, monocytosis/macrophage/lymphocytosis activation, ventriculitis, and choroid plexus lesions. However, CPZ monotherapy provided no histological benefactor. Remarkably, CVSN@CPZ induced restoration of brain tissue architecture to near normal states with markedly diminished inflammation and intact architecture. This therapeutic advantage, importantly, was observed in both ETEC and SAU meningitis models (Figure 8M and Figure 9M).

In summary, our study systematically confirmed the potent therapeutic efficacy of CVSN@CPZ in ETEC and SAU meningitis mouse models. This nanocarrier strategy not only effectively inhibited bacterial growth and significantly improved survival, but also alleviated neuroinflammation, providing a multifaceted intervention for BM. These findings highlight the translational potential of nanodelivery systems in treating complex central nervous system infections and provide valuable theoretical and experimental support for future clinical research.

### 2.10 Mechanistic Insights Into the Therapeutic Effects of CVSN@CPZ in Bacterial Meningitis

To elucidate the underlying mechanisms of CVSN@CPZ therapy, IF analysis was conducted to systematically assess changes in the immune microenvironment and cellular phenotypes across four dimensions.

#### 2.10.1 Epithelial Barrier Integrity and Inflammatory Infiltration

During inflammation, the expression of the tight junction protein ZO‐1 is markedly reduced, indicating impairment of BBB integrity [34]. Both the model and CPZ groups exhibited pronounced downregulation of ZO‐1, whereas treatment with CVSN@CPZ significantly preserved ZO‐1 levels, thereby effectively mitigating meningeal barrier disruption (Figure 8N and Figure 9N). Meanwhile, the levels of Cd11b (a marker of activated myeloid cells), Ly6G (a marker of neutrophils), and MPO (myeloperoxidase) were significantly elevated in the model and CPZ groups, suggesting that excessive infiltration of myeloid cells and MPO activation aggravated tissue injury. By contrast, CVSN@CPZ treatment markedly reduced these indices, limiting inflammatory cell accumulation and preventing secondary damage (Figure 8O and Figure 9O).

#### 2.10.2 Macrophage Polarization and Inflammatory Regulation

In the model and CPZ groups, expression of F4/80 (macrophage marker) and CD86 (pro‐inflammatory M1 phenotype marker) was strongly upregulated, while CD206 (anti‐inflammatory, reparative M2 phenotype marker) was suppressed, indicating an aggravated CNS inflammatory response. CVSN@CPZ treatment effectively downregulated CD86 while promoting CD206 expression, thereby driving macrophage polarization from the M1 toward the M2 phenotype, facilitating inflammation resolution and tissue repair (Figure 8P and Figure 9P).

#### 2.10.3 Apoptosis and Signaling Pathway Modulation

Caspase‐3, a critical executor of apoptosis, was significantly elevated in the model and CPZ groups, leading to excessive neuronal apoptosis. CVSN@CPZ treatment markedly inhibited Caspase‐3 activation, thus reducing neuronal loss (Figure 8Q and Figure 9Q). Furthermore, the Myd88 adaptor protein and NF‐κB signaling pathway were hyperactivated in the model and CPZ groups, perpetuating inflammatory cytokine release. CVSN@CPZ effectively attenuated this pathway, thereby curtailing sustained neuroinflammation (Figure 8R and Figure 9R) (Figures S36–S41).

#### 2.10.4 TLR Signaling and Amplification of Inflammation

Toll‐like receptors (TLRs), as central components of the innate immune system, recognize pathogen‐associated molecular patterns (PAMPs) and damage‐associated molecular patterns (DAMPs), thereby activating downstream immune responses [35]. TLR4 recognizes LPS from the *Escherichia coli* cell wall as well as DAMPs derived from *Staphylococcus aureus*, amplifying the inflammatory cascade [36]; whereas TLR9 detects bacterial DNA sequences [37]. Immunohistochemistry (IHC) analyses revealed significant activation of both TLR4 and TLR9 in the BM model. In contrast, CVSN@CPZ treatment effectively suppressed stimulatory signals—including lipoproteins, LPS, flagella, pathogen‐derived DNA, and host cfDNA—thereby attenuating the amplification of inflammatory responses. Further analysis demonstrated that the release of downstream proinflammatory cytokines TNF‐α and IL‐1β was markedly reduced in the treatment group, leading to substantial alleviation of the overall inflammatory response (Figure 8S and Figure 9S) (Figures S42 and S43).

Collectively, CVSN@CPZ exerts multifaceted immunomodulatory effects that substantially improve the pathological state of BM. Therapeutic benefits include preservation of BBB integrity, reduction of excessive myeloid infiltration and associated tissue damage, and promotion of macrophage polarization from pro‐inflammatory M1 to anti‐inflammatory M2 phenotypes, accelerating inflammation resolution and tissue repair. At the molecular level, CVSN@CPZ inhibited the TLR4/TLR9‐Myd88‐NF‐κB axis, leading to significant downregulation of TNF‐α and IL‐1β, thereby breaking the cycle of excessive inflammation. Moreover, suppression of neuronal apoptosis further protected CNS function. Through this multi‐layered synergistic regulation, CVSN@CPZ effectively restored immune homeostasis and promoted recovery toward a near‐normal state.

## 3 Discussion

Bacterial meningitis is a severe infectious disease of the central nervous system, characterized by acute onset, rapid progression, and a high mortality [38]. Although traditional antibacterial treatment has alleviated the infection to some degree, the BBB greatly limits the penetration efficiency of most antibiotics into the CNS [39]. This makes drug concentrations at the site of infection frequently inadequate and results in poor therapeutic effect. Moreover, traditional treatments demand a major systemic dose associated with adverse events and associated antibiotic resistance [40]. Thus, the emergence of a new and effective drug delivery system has become one of the key challenges of science.

Through this work, we developed and explored virus‐like mesoporous silica nanoparticles as a new drug delivery medium for CPZ targeting BM, which could be addressed as a therapeutic modality. Our data show the realization of this system that is not only a notable progress in the synthesis and structural design of the material, but we also get a large advantage to cross the BBB, the antibacterial efficacy in vitro, and the in vivo ability of the therapeutic drug to be increased. Taken together, these findings suggest this platform as a new route to address existing gaps in BM therapies.

We synthesized virus‐like mesoporous silica nanoparticles and improved their biocompatibility and brain‐targeting ability through the chemical grafting of L‐ala. The biomimetic viral surface had distinctive advantages for nanodrug delivery, such as the high surface roughness and homogeneous mesoporous architecture that enhanced the interaction with cellular membranes and endocytosis. Bio‐TEM and microglia‐ and astrocyte‐specific uptake assays showed that CVSN could effectively penetrate the BBB and be internalized by immune‐associated cells in the brain. This property in particular holds relevance for BM therapy, in which microglia and astrocytes are not just regulators of inflammatory responses but also essential participants in local distribution and metabolism of drugs [41]. In contrast with classic linear nanoparticles, CVSN exhibited better brain‐targeting efficiency, which could likely be due to the synergistic activities of an enhanced virus‐imitating morphology and surface modification.

Building on this foundation, we developed a robust drug delivery system by loading CPZ into CVSN (CVSN@CPZ), which substantially improved drug release controllability and utilization efficiency. Pharmacokinetic analyses confirmed that this nano‐system efficiently crossed the BBB and enabled targeted release within the brain parenchyma, ventricles, and choroid plexus (CP). Notably the CP represents one of the most significantly affected sites in the pathophysiology of BM, where functional disruption directly impairs CSF circulation and immune surveillance [42]. Targeted release of CVSN@CPZ within the CP not only increased local drug concentration but also sustained antibacterial activity in the meninges via CSF circulation. This cross‐compartment and sustained release profile effectively addresses the long‐standing limitation of insufficient antibiotic distribution in the CNS and represents a promising strategy to optimize the treatment of BM.

A series of assays to systematically in vitro was conducted assess the antibacterial efficacy of CVSN@CPZ. The results demonstrated that this nano‐system significantly reduced MIC of CPZ, thereby enhancing antibacterial activity at lower dosages. Quantitative bacterial assays further showed that the bacterial load in the CVSN@CPZ‐treated group was markedly lower than that in the free CPZ group. Moreover, crystal violet and PI staining corroborated the augmented bactericidal effect and revealed concomitant disruption of bacterial membranes. Collectively, these findings indicate that this nano‐delivery platform not only increases the local effective concentration of the drug but also synergistically enhances its bactericidal effect through specific interactions between the nanoparticles and bacterial membranes. Previous studies have suggested that the surface properties of mesoporous silica nanoparticles promote bacterial membrane binding and perturb membrane permeability [43]. Our results are consistent with these observations and further highlight the unique contribution of virus‐like surface topography in amplifying antibacterial potency.

In our murine model of BM, CVSN@CPZ also demonstrated strong therapeutic effects. HE staining studies showed decreased neuroinflammation and tissue necrosis, as well as reduced proinflammatory cytokines and improved neurological outcomes. It seems that these benefits may result from not only prolonged drug release, but from multi‐dimensional immunomodulation. In particular, CVSN@CPZ maintained BBB structure by preserving ZO‐1 expression, alleviated secondary inflammatory damage via restriction of Cd11b^+^, Ly6G^+^ and MPO^+^ myeloid cell infiltration, and reinforced immune homeostasis by enhancing CD206^+^ M2 macrophage polarization and inhibiting CD86^+^ M1 hyperactivation. Additionally, CVSN@CPZ inhibited transduction of aberrant activity of the inflammatory signal‐generating TLR4/TLR9‐Myd88‐NF‐κB axis, resulting in diminished synthesis of proinflammatory cytokines (for example TNF‐α, IL‐1β), and suppression of caspase‐3‐dependent nerve death. Through the dual modulation of innate immune signaling and maintenance of neuronal viability, CVSN@CPZ exerted a therapeutic synergy that was reflected in enhanced effect in BM.

The novelty identified in this study is the rational integration of virus‐inspired design with mesoporous silica nanomaterials, which result in a targeted drug delivery system with controlled release and effective antibacterial action. In contrast to traditional antimicrobials, CVSN@CPZ helps promote drug bioavailability at the site of infection and lowers the dose and systemic exposure and thereby relieves the selective pressure for resistant strains and lowers the risk of antimicrobial resistance. These properties have key implications for the long‐term management of BM. Moreover, this virus‐mimetic nanoplatform could serve as a promising paradigm for the treatment of other CNS infectious diseases.

Despite the enhanced brain accumulation achieved by the CVSN‐based delivery system, several limitations should be acknowledged. The current formulation does not incorporate a brain‐ or disease‐microenvironment‐responsive gating mechanism for drug release. Although drug release was evaluated in PBS (pH 7.4) and artificial cerebrospinal fluid, and in vivo biodistribution studies demonstrated increased drug exposure in the brain following CVSN loading, drug release under physiological conditions and detectable distribution in peripheral tissues indicate that premature release and nonspecific off‐target exposure cannot be completely excluded. Future studies will therefore focus on integrating additional brain‐targeting strategies and/or disease‐microenvironment‐responsive release mechanisms into the CVSN platform to further improve spatially selective drug release and minimize systemic exposure.

## 4 Conclusions

The virus‐mimicking nanoparticles used in the study present enhanced BBB penetration on account of biomimetic construction and delivered efficiently to brain parenchyma and ventricles using targeted accumulation leading to the selective delivery of therapeutic agents. Compared with the free drugs, this delivery system not only significantly increased the bioavailability of CPZ in brain tissues but also allowed the targeted lesions in the CP to be well targeted. Moreover, the antibiotic‐loaded nanoparticles maintained sustained drug release across the meningeal surfaces, allowing prolonged and intense suppression of bacterial proliferation and dissemination at the sites of infection through the CSF circulation. Collectively, these findings represent novel routes for the highly specific delivery of antibacterial agents to the CNS and suggest that virus‐inspired nanoplatforms could become a next‐generation therapeutic strategy for BM.

## 5 Materials and Methods

### 5.1 Ethical Statement

Our study complied with all relevant ethical regulations. All animal experiments were approved by the Animal Ethics Committee of China Medical University (Approval No. CMU20240469). In accordance with the committee's requirements, mice were housed under specific pathogen‐free (SPF) conditions.

### 5.2 Animals, Cells, and Bacterial Culture

Male C57BL/6 mice (6‐8 weeks old, ∼20 g) were used in this study and received humane care. Animals were maintained at room temperature under a 12 h light/dark cycle with free access to food and water, and acclimatized for one week prior to experiments. Although only one sex was used in this study, we believe the findings are not restricted to a single gender.

BV2 cell culture: The culture medium was prepared with 45 mL high‐glucose DMEM supplemented with 5 mL 10% fetal bovine serum (FBS) and 500 µL penicillin‐streptomycin (100 U mL^−1^). Prior to resuscitation, a water bath was preheated to 37°C. BV2 cells (<20 passages) were rapidly retrieved from a −80°C freezer or liquid nitrogen tank, gently shaken in the water bath, and thawed within 2 min. The suspension was centrifuged at 1500 rpm for 5 min. After surface sterilization of the cryovial with 75% ethanol, the supernatant was discarded in a biosafety cabinet, and the pellet was resuspended in 1 mL fresh medium. Cells were transferred to a T25 flask and cultured at 37°C with 5% CO_2_. After 24 h, adherent cells were observed and the medium was replaced. When cell confluence reached 70–90%, BV2 cells were digested with 0.25% trypsin‐EDTA and passaged at a 1:2–1:4 ratio. Cells were seeded into 25 cm^2^ or 75 cm^2^ flasks, or six‐well plates for subsequent use.

Bacterial culture: Bacteria were cultured in solid LB medium containing 5 g L^−1^ yeast extract, 10 g L^−1^ NaCl, 10 g L^−1^ tryptone, and 15 g L^−1^ agar at 37°C in a CO_2_ incubator (Thermo Scientific BB 150, MA, USA). The medium was sterilized at 120°C for 20 min. For liquid LB medium, agar was omitted under the same conditions.

### 5.3 Reagents

Cetyltrimethylammonium bromide (CTAB), 3‐aminopropyltriethoxysilane (APTES), tetraethoxysilane (TEOS), and L/D‐Boc‐Ala were purchased from Aladdin Biotech (Shanghai, China). 1‐(3‐Dimethylaminopropyl)‐3‐ethylcarbodiimide hydrochloride (EDCI), 1‐hydroxybenzotriazole (HOBT), and trifluoroacetic acid (TFA) were purchased from Rhawn Chemical Technology (Shanghai, China). Rhodamine B isothiocyanate (RITC) was obtained from Bailingwei Technology (Beijing, China). CPZ was purchased from Enzo Biochem (Shanghai, China). All chemicals were of analytical grade and used without further purification.

### 5.4 Synthesis of CVSN

Preparation of VSN: Briefly, 40 mL deionized water was added into a 250 mL round‐bottom flask, followed by 1 g cetyltrimethylammonium bromide (CTAB) and 0.18 g triethanolamine (TEA). The mixture was stirred in a water bath at 60°C for 2 h. A mixed solution of cyclohexane and tetraethoxysilane (TEOS) (4:1, v/v; 20 mL in total) was then added dropwise into the flask and the reaction was continued under stirring for 48 h. The flask was subsequently transferred to an oil bath at 98°C and stirred for another 24 h. After completion, the product was alternately washed with deionized water and absolute ethanol, followed by drying. To remove the template, 600 mg of the obtained nanoparticles were dispersed in 100 mL methanol containing 4 mL hydrochloric acid and refluxed at 70°C for 12 h. The particles were then washed, dried at 45°C, and collected as the final product (VSN).

Preparation of CVSN: VSN was first functionalized with amino groups via APTES modification. Specifically, 100 mg VSN and 300 µL APTES were dispersed in 30 mL absolute ethanol and stirred in a 50°C water bath for 24 h. The product was repeatedly washed with ethanol, centrifuged, and dried to yield amino‐modified VSN (AVSN). Subsequently, chiral molecules of L‐alanine were conjugated onto AVSN via an acylation reaction. In detail, 20 mg AVSN was dispersed in 5 mL dimethyl sulfoxide (DMSO). Then, 82.34 mg EDCI, 58.04 mg HOBT, and 81.27 mg Boc‐L‐alanine were added, and the mixture was stirred at room temperature for 48 h. The product was centrifuged, washed alternately with water and ethanol, and dried to obtain Boc‐protected CVSN (Boc‐CVSN). The Boc group was removed using trifluoroacetic acid (TFA). Briefly, 15 mg Boc‐CVSN was dispersed in 15 mL DMSO, followed by the addition of 6.53 mL TFA. The mixture was stirred at room temperature for 3 h. The product was centrifuged, washed thoroughly with ethanol, and dried to obtain the final CVSN.

Preparation of RITC‐CVSN: To prepare RITC‐CVSN, 100 mg rhodamine B isothiocyanate (RITC) and 100 µL APTES were dissolved in 2 mL absolute ethanol and stirred at room temperature for 24 h to form RITC‐APTES. Separately, 100 mg CVSN was dispersed in 5 mL ethanol, followed by the addition of 20 µL RITC‐APTES. The mixture was wrapped in foil and stirred at room temperature for 24 h. The resulting product was repeatedly washed with ethanol, dried, and collected as RITC‐CVSN.

Preparation of MSN: First, 30 mL of absolute ethanol was added to 100 mL of deionized water to form a homogeneous reaction solvent. Then, 0.8 g of CTAB was dissolved in the mixture under magnetic stirring to generate a stable micellar template. After the solution became clear, 0.8 mL of ammonia was added, and the system was stirred for another 10 min to ensure uniformity. Subsequently, 3 mL of TEOS was added dropwise. Under alkaline conditions, TEOS gradually underwent hydrolysis and condensation, leading to the formation of an ordered mesoporous silica framework around the CTAB micelles. The reaction mixture was stirred at room temperature for 4 h and then aged for 24 h. The resulting precipitate was collected by centrifugation or vacuum filtration and dried to obtain the MSN precursor.

To remove the CTAB template, the dried sample was dispersed in 100 mL of methanol containing 4 mL of hydrochloric acid, followed by reflux at 70°C for 12 h. After the reaction, the sample was collected by centrifugation or vacuum filtration and dried.

### 5.5 Fluorescence Stability Evaluation

To assess the fluorescence stability of CVSN, RITC‐CVSN was dispersed in PBS (pH 7.4) and aCSF, and incubated for 0, 2, 6, 12, and 24 h. The fluorescence spectra of the supernatant were measured using a microplate reader.

### 5.6 Nanoparticle Characterization

The morphology and spike‐like surface structures of nanoparticles (NPs) were examined using transmission electron microscopy (TEM, Tecnai G2‐F30, FEI, USA) and scanning electron microscopy (SEM, SUPRA 35, ZEISS, Germany). Fourier transform infrared (FTIR) spectra were recorded in the range of 400–4000 cm‐1sing a spectrometer (Spectrum 1000, Perkin Elmer, USA) to analyze the chemical composition of the nanodrugs. X‐ray photoelectron spectroscopy (XPS, ESCALAB 250Xi, Thermo Fisher Scientific, USA) was employed to determine the elemental composition (C, N, O, and Si) of VSN and CVSN. The surface charge characteristics and colloidal stability of the nanoparticles were evaluated in ethanol using a zeta potential analyzer (Zetasizer Nano ZS, Malvern, UK). Circular dichroism (CD) spectra were recorded with a multifunctional CD spectrometer (MOS‐500, Bio‐Logic, France) to detect conformational changes in chirality induced by L‐alanine grafting onto the VSN surface.

Nitrogen adsorption‐desorption isotherms were measured using a gas adsorption analyzer (V‐Sorb 2800P, Gold APP, China) to determine the pore structure of nanoparticles. The specific surface area (SBET) and average pore volume (Vt) of CVSN and CVSN@CPZ were calculated by the Brunauer‐Emmett‐Teller (BET) and Barrett‐Joyner‐Halenda (BJH) methods, respectively. Small‐angle X‐ray scattering (SAXS) profiles of CVSN and intermediates were obtained with an X‐ray diffractometer (DX‐2700X, China) to characterize scattering in the low‐angle region. The mesoporous structure of CVSN was further analyzed by BJH pore size distribution (V‐Sorb 2800P, China). Thermogravimetric analysis (TGA, TG 209 F3, Germany) was performed to evaluate the thermal stability and its evolution throughout the synthesis process.

### 5.7 Long‐Term Storage and Serum‐incubation Stability of CVSN

Freshly synthesized CVSN were characterized by Fourier‐transform infrared (FTIR) spectroscopy as the baseline sample. For long‐term storage stability assessment, an aliquot of CVSN was stored for 1 year and subsequently analyzed by FTIR. For serum‐incubation stability assessment, CVSN were incubated at 37°C for 7 days. The nanoparticles were subsequently recovered by centrifugation, washed to remove residual serum components. FTIR spectra were then recorded over 400–4000 cm^−^^1^ using the same acquisition conditions as those used for freshly synthesized CVSN.

### 5.8 Interfacial Properties of CVSN

The octanol‐water partition coefficient (Pow) was determined by a shaking‐flask method to evaluate the distribution of nanodrugs between hydrophilic and lipophilic phases. Prior to measurement, saturated solutions were prepared by shaking 100 mL n‐octanol with 300 mL water at 30°C for 24 h. RITC‐VSN and RITC‐CVSN were dispersed in 1 mL octanol‐saturated water, followed by addition of 4 mL water‐saturated octanol. The mixture was vortexed for 3 min and shaken at 30°C for 1 h. Fluorescence intensities in the aqueous and organic phases were measured using a microplate reader (Infinite 200 Pro, Tecan Austria GmbH, Austria). The partition coefficient was calculated according to the following equation:

(1) Pow=Fo/Fw

where *Fw* represents the equilibrium fluorescence intensity of NPs in the aqueous phase and Fo denotes that in the organic phase.

### 5.9 Degradation of CVSN

Accurately weighed 10 mg of CVSN and VSN were placed into separate 5 mL EP tubes, followed by the addition of 5 mL PBS (pH 7.4) and aCSF, respectively. The tubes were sealed and incubated in a thermostatic water bath shaker at 37°C with a shaking speed of 100 rpm. At predetermined time intervals, the samples were collected, centrifuged, and the residual carriers were gently rinsed with deionized water, dried, and weighed. The degradation rate (*WLt*) was subsequently calculated. Here, *W*_0_ represents the initial weight of the EP tube after labeling, *W_i_* denotes the total weight of the EP tube containing the mesoporous silica samples at the starting point, and *W_t_* indicates the total mass obtained after centrifugation and drying at different sampling time points.

(2)

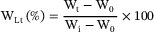




### 5.10 Biosafety Evaluation

Cytotoxicity: The cytotoxicity of the nanocarriers was evaluated using the CCK‐8 assay. Precisely weighed amounts of VSN and CVSN were dissolved in 1 mL DMSO and subsequently diluted with DMEM to prepare solutions of different concentrations (0, 0.01, 0.05, 0.1, 0.5, 1, 5, 10, and 50 µg mL^−1^). BV2 cells in logarithmic growth phase were harvested by trypsinization and centrifuged at 1500 rpm for 5 min. The cell suspension was seeded into 96‐well plates at a density of 5 × 10^4^ cells well^−1^ in 200 µL medium (DMEM supplemented with 10% fetal bovine serum and 1% penicillin‐streptomycin), followed by incubation at 37°C under 5% CO_2_ for 24 h. Thereafter, the culture medium was replaced with 200 µL nanocarrier solutions of different concentrations and incubated for another 24 h. Then, 20 µL of CCK‐8 solution (5 mg mL^−1^) was added to each well, and cells were incubated for an additional 4 h. The optical density (OD) at 570 nm was measured using a microplate reader, with blank wells used for zero adjustment. Cell viability (*Cv*) was calculated according to the following formula:

(3) $$C_{v}\left(\%\right)=\frac{A_{e}-A_{b}}{A_{c}-A_{b}}\times 100$$

where *A_e_ A_b_*, and *A_c_* represent the OD values of the experimental, blank, and control groups, respectively.

Hemocompatibility: The hemolysis assay was performed to assess blood compatibility. Whole blood was collected from healthy C57 mice, centrifuged, and washed to obtain red blood cells (RBCs). RBCs were suspended in sterile saline to prepare a 2% (v/v) suspension. Equal volumes (2 mL) of RBC suspension and CVSN saline suspensions of different concentrations were mixed and incubated at room temperature for 4 h. After centrifugation, the absorbance of the supernatant was measured at 540 nm. The hemolysis ratio (*Hr*) was calculated using the following formula:

(4) $$H_{r}\left(\%\right)=\frac{A_{s}-A_{n}}{A_{p}-A_{n}}\times 100$$

where *A_s_*, *A_p_*, and *A_n_* denote the absorbance values of the sample, positive control, and negative control groups, respectively.

Protein Adsorption: The protein adsorption behavior of CVSN in physiological environments was simulated using a bovine serum albumin (BSA) adsorption assay. A BSA stock solution (0.5 mg mL^−1^) was prepared in PBS (pH 7.4). Ten milligrams of CVSN were dispersed in 5 mL PBS, sonicated, and subsequently mixed with 5 mL BSA stock solution. The suspension was incubated in a shaking water bath at 37°C for 6 h. After centrifugation at 5000 rpm for 5 min, the supernatant was filtered through a 0.22 µm membrane, and the absorbance was measured at 280 nm. The adsorption capacity (*q*) was calculated according to the following formula:

(5) $$q\;(\%)=\frac{C_{0}-C_{a}}{m}\times \;100$$

where *C_0_* and *C_a_* are the initial and equilibrium concentrations of BSA, and *m* is the mass of nanocarriers.

Toxicity In Vivo : To evaluate systemic toxicity, six wild‐type mice were randomly divided into the control group and CVSN group (*n* = 3 per group). The body weight of each mouse was recorded before treatment. Mice in the CVSN group received a tail vein injection of CVSN solution (100 mg kg^−1^), while control mice received an equivalent volume of saline. Body weight was monitored every two days, and growth curves were plotted. After 15 days, blood samples were collected from the retro‐orbital sinus. Whole blood was collected into EDTA‐2Na tubes for routine hematology analysis, and additional samples were collected into heparinized tubes, centrifuged at 8000 rpm for 5 min, and subjected to biochemical analysis.

After blood collection, mice were euthanized, and organs were excised and weighed to calculate the organ‐to‐body weight ratio (organ index). Tissues were fixed in 4% paraformaldehyde, dehydrated, embedded in paraffin, sectioned at ∼5 µm thickness, and stained with HE. Histological morphology and pathological features were examined under an optical microscope.

### 5.11 Intravenous Absorption and Biodistribution

Eighteen male C57 mice (8 weeks old) were randomly divided into six groups (n = 3), with unrestricted access to food and water. A suspension of RITC‐CVSN in physiological saline (3 mL, 100 mg kg^−1^ body weight) was administered intravenously. At predetermined time points (1, 2, 4, 8, 12, and 24 h), the mice were perfused with PBS followed by paraformaldehyde solution. The heart, liver, spleen, lung, kidney, and brain were excised and fixed in paraformaldehyde‐containing EP tubes. Fluorescence images were acquired using a small animal imaging system (Carestream FX PRO, Bruker/Carestream, USA; Ex/Em: 570/625 nm), and the average fluorescence intensity of each organ was quantified via ROI analysis. Brain fluorescence intensity was further examined under a stereomicroscope (M205FA, Leica, Germany).

To quantitatively evaluate the absorption and biodistribution of CVSN, mice received an intravenous injection of CVSN (100 mg kg^−1^) and were sacrificed at 2, 6, and 24 h post‐injection (*n* = 3 per time point). Major organs, including the heart, liver, spleen, lung, kidney, and brain, were harvested, weighed, homogenized, and digested in 3 mL nitric acid at room temperature. The digested samples were transferred to a 64‐well heating apparatus, heated at 90°C for ∼1 h until no solid residue remained, and subsequently raised to 120°C for acid evaporation until yellow fumes were no longer observed. The digests were diluted to 15 mL with ultrapure water, filtered, and further diluted at a 1:1 ratio. Silicon content in plasma and organ samples was quantified using ICP‐MS (iCAP RQ, Thermo Scientific, USA) with calibration curves.

For brain tissue evaluation, RITC‐CVSN were intravenously administered into the leg vein of C57 mice. After 2 h, lectin was injected, followed by perfusion. The brains were sectioned at 100 µm thickness using a vibratome (VT1000S, Leica, Germany) at a speed of 1 mm s^−1^. Whole‐brain scanning across three fluorescence channels was performed using a multipurpose tissue scanner (Aperio Versa 8, Leica, Germany), and local Z‐stack imaging was conducted with a laser confocal microscope (Nikon AXR, Nikon, Tokyo, Japan). Fluorescence intensity was quantified using ImageJ software.

### 5.12 Uptake by Microglia in the Brain

Bio‐TEM was used to examine the distribution of nanoparticles (NPs) across the BBB. Mice were administered CVSN (100 mg kg^−1^) via tail vein injection, followed by perfusion with PBS and PFA at 2 h post‐injection, and brain samples were processed using standard bio‐TEM procedures.

To further confirm phagocytosis of CVSN by microglia, experiments were conducted in transgenic mice expressing microglia‐specific spontaneous fluorescence (Cx3cr1‐GFP, Jackson Laboratory, USA). Mice were intravenously injected with RITC‐CVSN (100 mg/kg) and sacrificed at 6 h post‐injection. Brain tissues were sectioned and mounted, and the colocalization of microglia with fluorescent nanoparticles was assessed using CLSM.

### 5.13 Uptake by Microglia In Vitro

When microglial cells reached 60%–80% confluence, they were digested and centrifuged at 1500 rpm for 5 min. After counting, cells were seeded onto coverslips placed in six‐well plates at a density of 5 × 10^5 cells per well and cultured for 24 h. Once the cell density on coverslips reached 60%–80%, culture medium containing VSN or CVSN was added, and the cells were incubated for 24 h. BV‐2 cells were then washed three times with PBS, fixed in 4% paraformaldehyde for 30 min, washed again, and mounted with DAPI‐containing mounting medium. Fluorescence images were acquired using CLSM.

### 5.14 CPZ Loading and Calculation

Drug‐loaded carriers were prepared using a solvent evaporation method. A CPZ‐acetone solution (10 mg mL^−1^) was first prepared. CVSN (20 mg) were mixed with 2 mL of the CPZ solution under stirring to achieve a drug‐to‐carrier ratio of 1:1 (w/w). After 24 h of stirring with an open cap to allow complete solvent evaporation, the resulting CPZ@CVSN drug‐loaded carriers were obtained by drying.

Determination of drug loading: A standard calibration curve was established using CPZ solutions of different concentrations in PBS. Precisely weighed 5 mg of CPZ@CVSN was placed into a 100 mL volumetric flask and diluted with PBS. After ultrasonication for 1 h, the solution was filtered through a 0.22 µm microporous membrane, and the absorbance at 228 nm was measured. The drug loading content (*Cdl*) and encapsulation efficiency (*Edl*) were calculated using Equations (6) and (7):

(6) $$C_{dl}\;(\%)=\frac{W_{D}}{W_{T}}\times \;100$$

(7) $$E_{dl}\;(\%)=\frac{W_{D}}{W_{TD}}\times \;100$$

where *W_D_* is the mass of drug encapsulated within the carrier pores, *W_TD_* is the total drug mass, and *W_T_* is the combined mass of drug and carrier.

### 5.15 Release of CPZ In Vitro

In vitro release was evaluated following the small‐cup method described in the Chinese Pharmacopoeia (2020, Part IV). The test was conducted at (37 ± 0.5) °C with a paddle speed of 100 rpm. Precisely weighed 10 mg of free CPZ and an equivalent CPZ‐loaded CVSN formulation (containing 10 mg CPZ) were separately added into 250 mL of release medium (PBS or ACSF). At predetermined time points (0, 5, 10, 15, 30 min, 1, 1.5, 2, 3, 4, 6, and 12 h), 3 mL of sample was withdrawn and replaced with fresh medium to maintain a constant volume. After filtration, absorbance was measured at 228 nm using a UV–Vis spectrophotometer.

### 5.16 Construction of an In Vitro BBB Model Using Transwell Inserts

1. Revival, Culture, and Passaging of bEnd.3 Cells. Thaw and culture bEnd.3 cells under standard conditions. Monitor cell morphology and proliferation to ensure optimal growth. Once the cells reach an appropriate density, aspirate the growth medium and replace it with serum‐free medium to induce serum starvation for 24 h. Digest the cells with trypsin and determine the cell density using a hemocytometer or automated cell counter.
2. Humidification of the Culture Plate. To minimize edge effects and reduce medium evaporation, add 1 mL of sterile PBS to each of the 16 peripheral wells of a 24‐well plate. Cover the plate with sealing film to maintain humidity.
3. Coating of Transwell Inserts. Prepare a coating solution of collagen type IV (50–100 µg mL^−1^) and add it to the upper chamber of each Transwell insert. Incubate the inserts at 37°C for 1–2 h or at 4°C overnight. After incubation, gently rinse the inserts three times with PBS to remove unbound collagen. Carefully aspirate any residual liquid using a pipette tip held against the wall of the insert to avoid disrupting the coating.
4. Cell Seeding and Culture. Seed 200 µL of bEnd.3 cell suspension (1 × 10^5^ cells per insert) into the apical chamber of each coated Transwell insert. Add 800 µL of complete culture medium to the basolateral compartment. Gently agitate the plate to distribute the cells evenly and place it in a humidified incubator at 37°C with 5% CO_2_. Replace the medium in both chambers every 48 h. Continue the culture for 15–20 days until the cells form a confluent and tight monolayer.
5. Measurement of Transendothelial Electrical Resistance (TEER). Calibrate the TEER measurement system in advance (zero the blank electrode). Remove the Transwell plate from the incubator and allow it to equilibrate at room temperature for 20 min. Insert the electrodes vertically into the apical and basolateral compartments, ensuring they do not touch the membrane surface. Record the electrical resistance of each well three times and calculate the mean value. A stable TEER value exceeding 15 mV indicates the successful formation of a functional barrier.

### 5.17 In Vitro Assessment of Drug and Carrier Permeation across a Monolayer Membrane

CPZ and CVSN@CPZ were added to the apical chamber of a monolayer. After 2 h, the basolateral medium was collected, and the CPZ content was quantified by HPLC. In parallel, RITC‐CVSN was introduced into the apical chamber, and after 2 h, Z‐stack images of the monolayer were acquired using CLSM. The fluorescence intensity per unit area was subsequently quantified using ImageJ software.

### 5.18 Pharmacokinetics

To investigate the in vivo bioavailability of CVSN@CPZ, C57 mice were randomly divided into two groups (*n* = 3). Mice received tail vein injections of either a CPZ solution (100 mg kg^−1^) or an equivalent dose of CPZ released from CVSN@CPZ (100 mg kg^−1^). Blood samples were collected at multiple time points within 2 h post‐injection. Plasma proteins were precipitated using acetonitrile: 80 µL plasma was mixed with 250 µL acetonitrile, vortexed for 1 min, and centrifuged for 10 min at 15 000 rpm. A 100 µL aliquot of supernatant was collected, centrifuged again for 5 min, and 20 µL of the final supernatant was injected into HPLC for analysis. Separation was performed on a Diamonsil C18 column (Ser. No. 8034287, 5 µm, 250 × 4.6 mm) with a C18 guard column, using a mobile phase flow rate of 1 mL min^−1^ at 25°C. Pharmacokinetic parameters were calculated using DAS 2.0 software.

For tissue distribution analysis, organ homogenates and diluted CSF samples were prepared and processed using the same method.

### 5.19 Antibacterial Activity In Vitro

To determine whether CVSN@CPZ exhibits antibacterial activity comparable to free CPZ, MIC was first measured using a standard drug sensitivity assay. Three groups (CVSN, CPZ, and CVSN@CPZ; *n* = 3 per group) were tested. *Escherichia coli* or *Staphylococcus aureus* suspensions (2 × 10^4 CFU/mL) were prepared by serial dilution using a turbidimeter. Equal volumes (50 µL) of drug solutions at different concentrations and bacterial suspensions were mixed, and 10 µL of the mixture was plated onto solid medium for 24 h incubation. The reduction in colony‐forming units (CFUs) and inhibition rates were calculated using the following equations:

(8) $$\Delta CFU=N_{0}\;-N_{t}$$

(9) $$R\;(\%)=\frac{N_{0}-N_{t}}{N_{0}}\times \;100$$

where *N*_0_ is the colony count in the control group and *N_t_* is the colony count under drug treatment.

To further evaluate bacterial membrane disruption, Bio‐TEM was performed. CPZ and CVSN@CPZ solutions at 2 × MIC were mixed with bacterial suspensions (1 × 10^8 CFU/mL) at equal volumes, incubated for 6 h, and processed for TEM imaging.

Biofilm inhibition assay: The anti‐biofilm activity of CVSN@CPZ was assessed using a crystal violet assay. Grouping, drug concentrations, and bacterial densities were the same as those in the MIC assay. Equal volumes of drug solution and bacterial suspension were mixed and incubated in 24‐well plates at 37°C for 24 h. Biofilms were stained with crystal violet for 30 min, washed with PBS, air‐dried, and imaged. The dye was then solubilized with 95% ethanol for 5 min, and absorbance was measured at OD570. The biofilm disruption rate (DR) was calculated as:

(10) $$DR=\left(\frac{OD_{control}-OD_{experimental}}{OD_{control}}\right)\times \;100$$

Live/dead bacterial staining: To quantify dead bacteria within biofilms, propidium iodide (PI) staining was used. Sterile coverslips were placed into 24‐well plates, inoculated with bacterial suspensions (1 × 10^4 CFU/mL), and incubated for 24 h to allow biofilm formation. After removing the supernatant, drug solutions of different concentrations diluted in LB medium were added, followed by another 24 h incubation. Biofilms were stained with PI, and fluorescence imaging was conducted using CLSM.

### 5.20 Antibacterial Pharmacodynamics of CPZ‐Loaded Carriers

The therapeutic efficacy of CVSN@CPZ was evaluated using a BM mouse model. Briefly, mice were anesthetized with isoflurane at an induction concentration of 4% and a maintenance concentration of 1.5%, positioned on a stereotaxic frame, and the cisterna magna was surgically exposed. A 5 µL suspension of E. coli or S. aureus (1 × 10^10 CFU/mL) was injected into the cisterna magna using a microinjection pump, with the needle retained in place for 10 min before closure.

Twenty‐one male C57 mice were randomly divided into seven groups: Sham, ETEC (BM), BM + CPZ, BM + CVSN@CPZ, SAU (BM), BM + CPZ, and BM + CVSN@CPZ. The Sham and BM groups received 5 µL of aCSF or bacterial suspension, respectively. At 24 h post‐surgery, mice in the BM + CPZ and BM + CVSN@CPZ groups received daily intravenous injections of CPZ or CVSN@CPZ (equivalent dose), while the Sham group received PBS, for 5 consecutive days.

Clinical evaluation and biochemical analysis: Mice were monitored daily for clinical symptoms. At the end of treatment, the first batch of mice was sacrificed, and blood samples were collected for plasma separation. Brains were dissected under sterile conditions, homogenized in PBS, and plated on LB agar for bacterial culture. Inflammatory cytokines in plasma and brain supernatants were quantified using ELISA.

Histopathology and immunoassays: A second batch of mice was perfused with PBS and paraformaldehyde. Brains were harvested, paraffin‐embedded, sectioned, and subjected to HE staining, IHC, and IF analysis.

### 5.21 Statistical Analysis

All statistical analyses were performed using GraphPad Prism 9. Each experiment was independently repeated three times. Statistical methods were selected according to dataset requirements and reported in Figure legends, including one‐way ANOVA, two‐way ANOVA, and two‐tailed t‐test. Exact *p* values were calculated at a significance threshold of 0.05. Data are presented as mean ± SEM.

## Author Contributions

Designing research studies: **T.D**., **H.L**., **G.L**., **M.L**.; Conducting experiments: **Q.X**., **X.Z**., **H.T**., **Y.L**., **X.F**., **M.X**., **Z.Z**., **L.Z**.; Acquiring data: **Q.X**., **X.Z**., **H.T**., **X.F**., **M.X**., **Z.Z**.; Analyzing data: **Q.X**., **X.Z**., **X.F**., **M.X**., **Z.Z**.; Writing the manuscript: **Q.X**., **T.D**., **H.L**., **G.L**.

## Funding

We acknowledge the foundation by the National Natural Science Foundation of China (No. 82370506), Xingliao Talent Program‐Young Top‐notch Talent Project (XLYC2503091), Liaoning Provincial Youth Science Foundation, Category B (2025‐YQ‐10), the Liaoning Province central guide local science and technology development fund program (No. 3190424019).

## Conflicts of Interest

The authors declare no conflicts of interest.

## Supporting information




**Supporting File**: advs78317‐sup‐0001‐SuppMat.docx.

## Data Availability

The data that support the findings of this study are available from the corresponding author upon reasonable request.
