# Supplementary material for: A Chiral Virus‐Like Nanoplatform That Broadly Mediates Antibiotic Delivery to the Brain for Bacterial Meningitis Therapy
Source: Adv Sci (Weinh). 2026 Oct 18:e78317. Online ahead of print. doi: 10.1002/advs.78317 (PMC13677075; doi:10.1002/advs.78317)
Supplement: Supplementary file 1 [file ADVS-9999-e78317-s001.docx]

**A chiral virus-like nanoplatform that broadly mediates antibiotic delivery to the brain for bacterial meningitis therapy**

Qianqian Xu^1, 2^†, Xuting Zheng^3†^, Huinan Tong^1†^, Ying Liu^1^†, Xiaoxi Fan^4^, Miao Xu^1^, Ziyan Zhang^1^, Li Zhang^1^, Mingyan Liu^1^*, Ting Du^1^*, Heran Li^1^*, Guojun Liu^2^*

^1^School of Pharmacy, China Medical University, Shenyang 110122, China

^2^ Department of Neurosurgery, the Fourth Affiliated Hospital of China Medical University, Shenyang 110032, China.

^3^Department of Infectious Disease, The First Hospital of China Medical University, Shenyang, 110001, China

^4^Department of Thoracic Surgery, The First Hospital of China Medical University, Shenyang 110001, China

†: These authors contributed equally

*: Co-Corresponding Authors

**Corresponding author:**

Guojun Liu

Department of Neurosurgery,

The Fourth Affiliated Hospital of China Medical University

Shenyang 110032, China.

[lgjsc0327@163.com](mailto:lgjsc0327@163.com)

Heran Li

School of Pharmacy,

China Medical University

Shenyang 110122, China

[liheranmm@163.com](mailto:liheranmm@163.com)

Ting Du

School of Pharmacy

China Medical University

Shenyang 110122, China

[duting_611@163.com](mailto:duting_611@163.com)

Mingyan Liu

School of Pharmacy

China Medical University

Shenyang 110122, China

myliu@cmu.edu.cn

**Supplementary figures and legends**

**Supplementary Figure 1.** Fourier transform‑infrared (FT‑IR) spectra of freshly synthesized CVSN, CVSN stored for 5 months, and CVSN incubated with serum for 7 days.

**Supplementary Figure 2.** (A) TEM and SEM images of MSN and DVSN. (B) Particle size distribution of MSN. (C) Particle size distribution of DVSN.

**Supplementary Figure 3.** (A) Thermogravimetric analysis (TGA) curve of NPs. (B) Fourier-transform infrared (FTIR) spectrum of NPs. (C) XPS spectrum of NPs. (D) Circular dichroism (CD) spectrum of NPs.

**Supplementary Figure 4.** Degradation profiles of CVSN in PBS (pH = 7.4) and aCSF. Data are presented as mean ± SEM, n=3, n refers to the number of biological replicates. Source data are available in the source data file.

**Supplementary Figure 5.** (A) Representative whole-body fluorescence imaging and (B) quantification of fluorescence intensity in major organs at different time points following intravenous administration of RITC-DVSN in mice. Data are presented as mean ± SEM, n=3, n refers to the number of biological replicates. (C) Representative whole-body fluorescence imaging and (D) quantification of fluorescence intensity in major organs at different time points following intravenous administration of RITC-VSN in mice. Data are presented as mean ± SEM, n=3, n refers to the number of biological replicates.

**Supplementary Figure 6.** Representative *ex vivo* fluorescence images of major organs collected at 1, 2, 4, 8, 12, and 24 h after intravenous administration of RITC-labeled (A) CVSN(0.5×L-Ala) and (B) CVSN(2×L-Ala). The heart, liver, spleen, lung, kidney, and brain were harvested at the indicated time points and subjected to fluorescence imaging. (C) Corresponding mean fluorescence energy per unit area in each organ was quantified by ROI analysis. Data are presented as mean ± SEM, n = 3 biological replicates.

**Supplementary Figure 7.** (A) Synthetic procedure of RITC-CVSN. (B-C) Fluorescence stability of RITC-labeled nanoparticles in PBS (pH = 7.4) and aCSF. Source data are available in the source data file.

**Supplementary Figure 8.** (A) Whole-scan images of brain sections. Blue: DAPI-stained nuclei; green: lectin-stained vasculature; red: RITC-DVSN/MSN. (B) Fluorescence intensity quantification. Data are presented as mean ± SEM, n=3, n refers to the number of biological replicates. *P<0.05. Two-sided unpaired t-test was performed.

**Supplementary Figure 9.** (A) CLSM images showing transvascular penetration of RITC-labeled DVSN and MSN at 2 h. Blue: DAPI; Green: Lectin; Red: RITC-DVSN/MSN. Yellow arrows indicate RITC-DVSN/MSN penetrating the blood–brain barrier (BBB) into the parenchyma. (B) BBB permeability of RITC- DVSN/MSN at 2h. Data are presented as mean ± SEM, n=3, n refers to the number of biological replicates. *P<0.05, **P<0.01. Two-sided unpaired t-test was performed.

**Supplementary Figure 10.** CLSM images of neuronal cells phagocytosing RITC-CVSN and statistical analysis of fluorescence intensity per unit area. Data are presented as mean ± SEM, n=3, n refers to the number of biological replicates. ****P < 0.0001. Two-sided unpaired t-test was performed.

**Supplementary Figure 11.**  (A) CLSM images showing the uptake of RITC-CVSN by microglia in Cx3cr1-GFP transgenic mice. (B) Line-scan analysis of colocalized fluorescence intensity. Source data are available in the source data file.

**Supplementary Figure 12.** Cytotoxicity of (A) VSN and (B) CVSN nanoparticles evaluated by the Cell Counting Kit-8 (CCK-8) assay after 24 h incubation with microglia. Data are presented as mean ± SEM, n=3, n refers to the number of biological replicates. One-way ANOVA post hoc Tukey’s test was performed. No significant difference observed.

**Supplementary Figure 13.** *In vitro* biosafety assessment of CVSN. (A) Representative images from hemolysis assay and (B) quantification of hemolysis rate. (C) Protein adsorption capacity test using bovine serum albumin. Data are presented as mean ± SEM, n=3, n refers to the number of biological replicates. Source data are available in the source data file.

**Supplementary Figure 14.** *In vivo* biosafety assessment of CVSN. (A) Experimental design for *in vivo* toxicity evaluation of CVSN. (B) Body weight gain ratio, (C) organ index, and (D) blood biochemical parameters in mice following intravenous administration of CVSN. Data are presented as mean ± SEM, n=3, n refers to the number of biological replicates. Two-way ANOVA with Bonferroni’s multiple comparisons test was performed (B-C). Two-sided unpaired t-test was performed (D).

**Supplementary Figure 15.** Hematological parameters from *in vivo* toxicity study. Data are presented as mean ± SEM, n=3, n refers to the number of biological replicates. Two-sided unpaired t-test was performed (D). No significant difference observed.

**Supplementary Figure 16.** Representative histopathological images of major organs from *in vivo* toxicity studies. Source data are available in the source data file.

**Supplementary Figure 17.** Molecular docking results of DVSN, along with statistical analysis of binding energies and the number of hydrogen bonds.

Supplementary Figure 18. Molecular docking analysis of L-arginine and D-arginine with proteins associated with different endocytic pathways. Representative molecular docking conformations of (A) L-arginine or (B) D-arginine with proteins associated with clathrin-mediated endocytosis, macropinocytosis, and caveolae-mediated endocytosis. Quantitative analysis of the predicted binding energies and numbers of hydrogen bonds between (C) L-arginine or (D) D-arginine and proteins associated with the three endocytic pathways.

Supplementary Figure 19. Representative molecular docking conformations of (A) L-arginine or (B) D-arginine with proteins associated with clathrin-mediated endocytosis, macropinocytosis, and caveolae-mediated endocytosis. Enlarged views illustrate the ligand-binding sites and intermolecular interactions, while surface representations show the spatial accommodation of L-arginine within the corresponding binding pockets. Quantitative analysis of the predicted binding energies and numbers of hydrogen bonds between (C) L-arginine or (D) D-arginine and proteins associated with the three endocytic pathways.

Supplementary Figure 20. Representative ex vivo fluorescence images of the heart, liver, spleen, lung, kidney, and brain collected at 1, 2, 4, 8, 12, and 24 h after intravenous administration of fluorescently labeled (A) VSN-L-Arginine or (B) VSN-D-Arginine; Quantitative analysis of fluorescence signals in the heart, liver, spleen, lung, kidney, and brain at different time points after administration of (C) VSN-L-Arginine or (D) VSN-D-Arginine.

Supplementary Figure 21. Representative *ex vivo* fluorescence images of the heart, liver, spleen, lung, kidney, and brain collected at 1, 2, 4, 8, 12, and 24 h after intravenous administration of fluorescently labeled (A) VSN-L-Cysteine or (B) VSN-D-Cysteine; Quantitative analysis of fluorescence signals in individual organs at different time points after administration of (C) VSN-L-Cysteine or (D) VSN-D-Cysteine.

Supplementary Figure 22. (A) Representative confocal laser scanning microscopy (CLSM) images of brain endothelial cells incubated with fluorescently labeled VSN-L-Cysteine, VSN-D-Cysteine, VSN-L-Arginine, or VSN-D-Arginine. Cell nuclei were stained with DAPI (blue), and RITC-labeled nanoparticles are shown in red. Merged images illustrate the intracellular distribution of the nanoparticles. (B) Quantitative analysis of intracellular RITC fluorescence intensity in the four treatment groups. Higher fluorescence intensities were observed for the L-configured cysteine- and arginine-functionalized nanoparticles than for their corresponding D-configured counterparts, supporting a chirality-dependent difference in endothelial cellular uptake.

**Supplementary Figure 23.** (A) Representative CLSM images of cells incubated with RITC-labeled VSN, CVSN(1×L-Ala), CVSN(0.5×L-Ala), or CVSN(2×L-Ala). Cell nuclei were stained with DAPI (blue), and RITC-labeled nanoparticles are shown in red. Scale bar, 50 μm. (B) Cellular uptake was quantified based on intracellular RITC fluorescence intensity. Data are presented as mean ± SEM, n = 3 biological replicates. ***P < 0.001.

**Supplementary Figure 24.** Uptake of RITC-DVSN by endothelial cells under PBS control and inhibition of clathrin-mediated endocytosis, macropinocytosis, and caveolin-dependent pathways, along with quantitative analysis of fluorescence intensity and uptake inhibition efficiency. Data are presented as mean ± SEM, n=3, n refers to the number of biological replicates.

**Supplementary Figure 25.** Uptake of RITC-MSN by endothelial cells under PBS control and inhibition of clathrin-mediated endocytosis, macropinocytosis, and caveolin-dependent pathways, along with quantitative analysis of fluorescence intensity and uptake inhibition efficiency. Data are presented as mean ± SEM, n=3, n refers to the number of biological replicates.

**Supplementary Figure 26.** Transmission electron microscopy (TEM) images showing the uptake of MSNs by microglia, astrocytes, and endothelial cells.

**Supplementary Figure 27.** Standard calibration curves of four antibiotics determined by Ultraviolet-Visible (UV) spectrophotometry. Source data are available in the source data file.

**Supplementary Figure 28.** Drug loading efficiency and encapsulation efficiency of four antibiotics. Data are presented as mean ± SEM, n=3, n refers to the number of biological replicates. Source data are available in the source data file.

**Supplementary Figure 29.** Drug release profiles of norfloxacin, levofloxacin, and imipenem in cerebrospinal fluid medium. Data are presented as mean ± SEM, n=3, n refers to the number of biological replicates. Source data are available in the source data file.

**Supplementary Figure 30.** Schematic illustration of (Cisterna magna) CM surgery for cerebrospinal fluid collection. Mice were anesthetized and securely positioned on a stereotaxic surgical platform. The skin and underlying muscle tissues over the foramen magnum were carefully dissected to expose the cerebellomedullary cistern. A pulled glass micropipette was then gently inserted into the cistern at a slight angle to minimize tissue damage. Cerebrospinal fluid (CSF) was slowly collected through the micropipette, after which the electrode was carefully withdrawn. The collected CSF samples were immediately processed and stored appropriately for subsequent analysis of drug concentrations using high-performance liquid chromatography (HPLC).

**Supplementary Figure 31.** Standard calibration curves of four antibiotics determined by HPLC.

**Supplementary Figure 32.** VSN and VSN@CPZ. Data are presented as mean ± SEM, n=3, n refers to the number of biological replicates.

**Supplementary Figure 33.** TEM images showing the inhibitory effect of VSN@CPZ on ETEC and SAU after co-incubation.

**Supplementary Figure 34.** Colony growth images of (A) ETEC and (D) SAU treated with different concentrations of VSN@CPZ. Quantification of colony reduction in (B) ETEC and (E) SAU treated with different concentrations of VSN@CPZ. Statistical analysis of inhibition rates of (C) ETEC and (F) SAU under the same treatments. Data are presented as mean ± SEM, n=3, n refers to the number of biological replicates.

**Supplementary Figure 35.** (A) Propidium iodide staining of Escherichia coli exposed to an antibiotic concentration of 4 μg/mL, and (C) propidium iodide staining of Staphylococcus aureus exposed to an antibiotic concentration of 128 μg/mL, showing the distribution of dead bacteria. Data are presented as mean ± SEM, n=3, n refers to the number of biological replicates. *P < 0.05, **P < 0.01, ***P < 0.001, ****P < 0.0001. One-way ANOVA post hoc Tukey’s test was performed (B). Two-sided unpaired t-test was performed (D).

**Supplementary Figure 36.** (A) ZO-1 staining (single channel). Blue: cell nuclei stained with DAPI; Green: ZO-1. (B) Ly6G, Cd11b, and MPO staining (single channel). Blue: cell nuclei stained with DAPI; Red: myeloid cells; Purple: myeloperoxidase (MPO) protein. (C) F4/80, Cd86, and CD206 staining (single channel). Blue: DAPI (cell nuclei); Red: F4/80 (macrophages); Green: CD86 (M1-like macrophage marker); Purple: CD206 (M2-like macrophage marker). The Sham cohort served as the common control for both the ETEC and SAU experiments; therefore, the representative Sham images shown here are also presented for the corresponding markers in Supplementary Fig. 38.

**Supplementary Figure 37.** (A) Caspase-3 staining (single channel). Blue: DAPI (cell nuclei); Red: Caspase-3 (apoptosis marker). (B) NF-κB and MyD88 staining (single channel). Blue: DAPI (cell nuclei); Red: NF-κB (inflammatory signaling marker); Green: MyD88 (innate immune signaling adaptor). The Sham cohort served as the common control for both the ETEC and SAU experiments; therefore, the representative Sham images shown here are also presented for the corresponding markers in Supplementary Fig. 39.

**Supplementary Figure 38.** (A) ZO-1 staining (single channel). Blue: cell nuclei stained with DAPI; Green: neutrophils. (B) Ly6G, Cd11b, and MPO staining (single channel). Blue: cell nuclei stained with DAPI; Red: myeloid cells; Purple: myeloperoxidase (MPO) protein. (C) F4/80, Cd86, and CD206 staining (single channel). Blue: DAPI (cell nuclei); Red: F4/80 (macrophages); Green: CD86 (M1-like macrophage marker); Purple: CD206 (M2-like macrophage marker). The Sham cohort is shared with Supplementary Fig. 36; therefore, the same representative Sham images are shown here for the corresponding markers.

**Supplementary Figure 39.** (A) Caspase-3 staining (single channel). Blue: DAPI (cell nuclei); Red: Caspase-3 (apoptosis marker). (B) NF-κB and MyD88 staining (single channel). Blue: DAPI (cell nuclei); Red: NF-κB (inflammatory signaling marker); Green: MyD88 (innate immune signaling adaptor). The Sham cohort is shared with Supplementary Fig. 37; therefore, the same representative Sham images are shown here for the corresponding markers.

**Supplementary Figure 40.** Quantitative analysis of immunofluorescence parameters for the ETEC group obtained using Image J. Data are presented as mean ± SEM, n=3, n refers to the number of biological replicates. *P < 0.05, **P < 0.01, ***P < 0.001, ****P < 0.0001. One-way ANOVA post hoc Tukey’s test was performed.

**Supplementary Figure 41.** Quantitative analysis of immunofluorescence parameters for the SAU group obtained using Image J. Data are presented as mean ± SEM, n=3, n refers to the number of biological replicates. *P < 0.05, **P < 0.01, ***P < 0.001, ****P < 0.0001. One-way ANOVA post hoc Tukey’s test was performed.

**Supplementary Figure 42.** ImageJ-based statistical results of the immunohistochemical positive rates of various markers in the ETEC group. Data are presented as mean ± SEM, n=3, n refers to the number of biological replicates. *P < 0.05, **P < 0.01, ***P < 0.001, ****P < 0.0001. One-way ANOVA post hoc Tukey’s test was performed.

**Supplementary Figure 43.** ImageJ-based statistical results of the immunohistochemical positive rates of various markers in the SAU group. Data are presented as mean ± SEM, n=3, n refers to the number of biological replicates. *P < 0.05, **P < 0.01, ***P < 0.001, ****P < 0.0001. One-way ANOVA post hoc Tukey’s test was performed.

**Supplementary Tables**

**Supplementary Table 1. Texture properties of CVSN and CVSN@CPZ**

| Sample | Surface Area (m^2^/g) | Pore Volume (cm^3^/g) | DR-MPD |
| --- | --- | --- | --- |
| CVSN | 246.5644 | 1.3648 | 2.3695 |
| CVSN@CPZ | 56.5933 | 0.4617 | 0 |

**Supplementary Table 2. Physicochemical properties of four antibiotics.**

| Drug | Molecular Weight (g/mol) | BCS Classification | Solubility | Optical Activity |
| --- | --- | --- | --- | --- |
| Cefoperazone | 667.66 | III | Freely soluble | Optically inactive |
| Norfloxacin | 319.33 | II | Sparingly soluble | Optically inactive |
| Levofloxacin | 361.37 | II | Sparingly soluble | Levorotatory |
| Imipenem | 299.35 | III | Freely soluble in water, but unstable | multiple chiral centers (5R, 6S) |

**Supplementary Table 3. Antibacterial spectrum and BBB penetration capacity of four antibiotics.**

| Drug | Class | BBB Penetration | Antimicrobial Spectrum |
| --- | --- | --- | --- |
| Cefoperazone | Cephalosporin | Limited (Inflammation-dependent) | Exhibits potent activity against Gram- (e.g., *E. coli*) and notable activity against some Gram+ (e.g., *S. aureus*). |
| Norfloxacin | Fluoroquinolone | Limited | Primarily against Gram-negative bacteria, e.g., E. coli. Also effective against Pseudomonas aeruginosa and some Gram+ bacteria, but with weaker potency. |
| Levofloxacin | Fluoroquinolone | Fair | Broad-spectrum: Potent activity against Gram-negative bacteria, atypical pathogens, and Gram-positive bacteria. |
| Imipenem | Carbapenem | Fair  (Inflammation-dependent) | Very broad-spectrum: Highly active against most Gram-positive bacteria, Gram-negative bacteria, and anaerobes. |

**Supplementary Table 4. HPLC conditions of four antibiotics loaded CVSN**

| Drug | Mobile phase and ratio | Detection wavelength |
| --- | --- | --- |
| Cefoperazone | 0.1%KH_2_PO_4_: Acetonitrile=25:75 | 254nm |
| Norfloxacin | 0.1%TFA: Acetonitrile=75:25 | 278nm |
| Levofloxacin | 0.1%TFA: Acetonitrile=75:25 | 295nm |
| Imipenem | 5mM Ammonium acetate: Acetonitrile =95:5 | 300nm |
